# Formalin Inactivation of Japanese Encephalitis Virus Vaccine Alters the Antigenicity and Immunogenicity of a Neutralization Epitope in Envelope Protein Domain III

**DOI:** 10.1371/journal.pntd.0004167

**Published:** 2015-10-23

**Authors:** Yi-Chin Fan, Hsien-Chung Chiu, Li-Kuang Chen, Gwong-Jen J. Chang, Shyan-Song Chiou

**Affiliations:** 1 Graduate Institute of Microbiology and Public Health, National Chung Hsing University, Taichung, Taiwan; 2 Department of Periodontology, School of Dentistry, National Defense Medical Center and Tri-Service General Hospital, Taipei, Taiwan; 3 College of Medicine, Tzu-Chi University, Hualien, Taiwan; 4 Arboviral Diseases Branch, Center for Disease Control and Prevention, Fort Collins, Colorado, United States of America; University of Texas Medical Branch, UNITED STATES

## Abstract

Formalin-inactivated Japanese encephalitis virus (JEV) vaccines are widely available, but the effects of formalin inactivation on the antigenic structure of JEV and the profile of antibodies elicited after vaccination are not well understood. We used a panel of monoclonal antibodies (MAbs) to map the antigenic structure of live JEV virus, untreated control virus (UCV), formalin-inactivated commercial vaccine (FICV), and formalin-inactivated virus (FIV). The binding activity of T16 MAb against Nakayama-derived FICV and several strains of FIV was significantly lower compared to live virus and UCV. T16 MAb, a weakly neutralizing JEV serocomplex antibody, was found to inhibit JEV infection at the post-attachment step. The T16 epitope was mapped to amino acids 329, 331, and 389 within domain III (EDIII) of the envelope (E) glycoprotein. When we explored the effect of formalin inactivation on the immunogenicity of JEV, we found that Nakayama-derived FICV, FIV, and UCV all exhibited similar immunogenicity in a mouse model, inducing anti-JEV and anti-EDII 101/106/107 epitope-specific antibodies. However, the EDIII 329/331/389 epitope-specific IgG antibody and neutralizing antibody titers were significantly lower for FICV-immunized and FIV-immunized mouse serum than for UCV-immunized. Formalin inactivation seems to alter the antigenic structure of the E protein, which may reduce the potency of commercially available JEV vaccines. Virus inactivation by H_2_O_2_, but not by UV or by short-duration and higher temperature formalin treatment, is able to maintain the antigenic structure of the JEV E protein. Thus, an alternative inactivation method, such as H_2_O_2_, which is able to maintain the integrity of the E protein may be essential to improving the potency of inactivated JEV vaccines.

## Author Summary

We demonstrated that formalin inactivation of Japanese encephalitis virus (JEV) alters the antigenic structure of the JEV envelope glycoprotein (E), in particular an epitope in domain III, and that this reduces the ability of the inactivated vaccine to elicit protective neutralizing antibodies. Ours and others’ previous studies have highlighted the importance of improving the immunogenicity of genotype III (GIII)-derived JEV vaccine in order to provide cross-protection against genotype I (GI) viruses, which are emerging and replacing GIII viruses in many JEV-endemic regions. Encouraging the wide use of live-attenuated or chimeric vaccines, such as SA14-14-2 or yellow-fever 17D/JEV vaccines, respectively, developing GI virus-derived inactivated or premembrane/E–containing, noninfectious virus-like particle (VLP) vaccines are two other possible ways to address this potential problem. In this exploratory study, we highlight an alternative inactivation method, such as H_2_O_2_ treatment, which may improve the antigenic stability and immunogenicity of JEV.

## Introduction

Japanese encephalitis virus (JEV), the most important etiological agent of viral encephalitis in Asian countries, causes regular outbreaks in eastern and southeastern Asia, India, and more recently in Australia [1,2]. Annually, 30,000 to 50,000 Japanese encephalitis (JE)-confirmed cases are reported in the JEV endemic areas, and 20% to 60% of symptomatic CNS infections are fatal [3–6]; 25% to 50% of symptomatic survivors have long-term neurological sequelae [7]. Asymptomatic JEV infection is about a thousand-fold higher than confirmed cases [8–10]. JEV is transmitted by virus-infected *Culex* mosquitos from inapparently infected viremic-amplifying hosts such as pigs or aquatic birds to symptomatic accidental hosts, such as horses and humans. Migratory birds have been implicated as the source of virus been introduced into new geographic regions, and associated with JE epidemics and replacement of genotype III (GIII)- with genotype I (GI)- JEV from southeast Asia to east Asia [11,12].

The genome of JEV consists of a ~11-kb, positive-sense, single-stranded RNA, which is translated and processed by viral and host proteases to three structural proteins—capsid, precursor membrane/membrane protein (prM/M) and envelope glycoprotein (E)—and seven nonstructural proteins (NS)—NS1, 2A, 2B, 3, 4A, 4B and 5. The mature virion consists of 180 E proteins forming 90 homodimers and 180 processed M proteins. The immature virion is formed by 60 E and prM hetero-trimers [13,14]. E protein is the most critical protein eliciting protective immunity in hosts after viral infection, offering critical protection in mice [15] and inducing protective antibodies in recovering humans [16]. The ectodomain of E protein can be separated into three structural domains: E domain I (EDI) to III (EDIII). The fusion peptide in EDII elicits group cross-reactive non- or low-neutralizing antibodies; EDIII, the receptor-binding domain, elicits potent type-specific neutralizing antibodies; and EDI, the center domain connecting EDII and EDIII, elicits complex cross-reactive high- or non-neutralizing antibodies after viral infection [16–18].

Vaccination remains the most effective strategy to control JE epidemics [19]. Live-attenuated and formalin-inactivated JEV vaccines are available for human use, but only live-attenuated vaccines are available for domestic animals, such as swine and horses. The first generation inactivated JEV vaccine, developed by BIKEN in Japan, was the mouse brain-derived, formalin-inactivated GIII Nakayama strain; manufacture of this vaccine has ceased since 2005 because of undesirable adverse effects [20]. Second generation tissue culture-derived, formalin-inactivated SA-14-14-2 vaccines are formulated with aluminum-hydroxide–adjuvant (IC51 or IXIARO). IC51 vaccine has been licensed for use in adult and children older than 2 months [21]. In addition, a live-attenuated JEV SA14-14-2 vaccine, developed in China, is used in some Asian countries such as China, India, and Nepal [22–24]. The vaccine effectiveness has been estimated to be 85% to 90% after two doses of inactivated Nakayama vaccine, and 91% after one dose of the live-attenuated SA14-14-2 vaccine [25–27]. Unlike the live-attenuated vaccine, the formalin-inactivated JEV vaccines require boost immunization to retain the protective neutralizing antibodies [22,28].

Significant numbers of JEV endemic countries still depend on the locally produced, mouse brain-derived formalin-inactivated GIII JEV vaccine to control JE epidemics [19]. Formalin is the chemical most commonly used for inactivation to manufacture viral vaccines such as hepatitis A virus, polio, influenza virus, rabies virus, and simian immunodeficiency virus [29–34]. Formalin reacts with amino acids of target proteins to form reversible Schiff-base adducts and non-reversible methylene bridges. It has also been used as isotopic agent to label protein by introducing isotope to specific amino acid and as a cell and tissue fixation agent. Formalin functions chemically when it is used to inactivate virus, and the chemical reaction may modify the antigenic structure of the virion [35,36]. It has been shown formalin inactivation alters antigenic properties and reduces the immunogenicity of vaccines, such as hepatitis A and B virus, polio virus, bovine herpes virus 1 and influenza virus in mouse models [37–41].

Formalin-inactivated JEV vaccine remains the most widely distributed vaccine used to control JE epidemics. However, the potential effects of formalin on the antigenic structure of JEV and the antibody profile elicited by this vaccine remain unclear. The use of a low concentration of formalin and short inactivation time can yield antigens capable of inducing high neutralizing titers in mice, but the association between these inactivation procedures and the alteration of antigenic structure of E and the antibody profile elicited by this vaccine remain undetermined [42].

In this study, we used a panel of E-specific, murine monoclonal antibodies (MAbs) to analyze the effect of epitope modification of JEV E protein in a formalin-inactivated commercial vaccine (FICV) and laboratory grown, formalin-inactivated GIII and GI viruses (FIV). We showed that formalin-inactivation, indeed altered the binding pattern of a JEV-derived, serocomplex cross-reactive neutralizing antibody, T16. Interestingly, antibodies recognizing formalin-modified epitope were significantly lower in titer and had weaker neutralizing activity in serum from mice vaccinated with FICV and FIV-Nakayama than with untreated control Nakayama virus (UCV-Nakayama). H_2_O_2_ inactivated JEV and was a superior approach that retained the antigenic reactivity of the virus with all tested MAbs including T16 as compared to conventional inactivation methods such as formalin and UV.

## Methods

### Ethics statement

Animal experiments were approved by the Institutional Animal Care and Use Committee (IACUC) of National Chung Hsing University, Taiwan (Approval No: 101–88), and performed according to a protocol, which adhered to principles in the Guide for the Care and Use of Laboratory Animals (NRC 2011) and meet the requirement in an Association for Assessment and Accreditation of Laboratory Animal Care (AAALAC).

The serum samples used in this study were collected from anonymous children who had received JEV vaccination and were without JEV infection during 2010; they were part of an already-existing collection housed at Tungs’ Taichung Metroharbor Hospital in Taichung. The clinical protocol was reviewed and approved by the institutional review board of the hospital (99006) for serum sample collection. Serum was recovered from blood after clotting and then centrifuged, and stored at -70°C until use.

### Cells and viruses

Vero, COS-1, and C6/36 cells (kindly provided from Dr. Chang GJ of US CDC, Fort Collins, CO) were grown in Dulbeco’s modified Eagle’s minimal essential medium (DMEM, Gibco) containing 5%, 10%, and 10% heat-inactivated fetal bovine serum (FBS, Gibco), respectively. BHK cells (kindly provided from Dr. Chen WJ of Chang Gung University, Taiwan) were grown in Minimum Essential Medium (MEM, Gibco) with 10% heat-inactivated FBS. The JEV vaccine strains used were the GIII strains Nakayama and SA14-14-2, naturally attenuated GIII T1P1 isolate [43] and GI circulating strain YL2009-4 [44].

### Reagents

The FICV used in this study was the mouse brain-derived, formalin-inactivated Nakayama virus vaccine manufactured by ADImmune Corp. in Taiwan. Monoclonal antibodies (MAbs) used for antigenic characterization were flavivirus group cross-reactive MAbs (4G2, 6B3B-3, 6B6C-1 and 23–2), JEV serocomplex cross-reactive MAbs (T16, 2B5B-3, 6B4A-10, 1B5D-1 and 7A6C-5) and JEV-specific MAbs (2H4 and 2F2) [45–47].

### Viral amplification, concentration and inactivation

Vero cells, infected with strains of JEV, namely Nakayama, SA14-14-2, T1P1, and YL2009-4, at a multiplicity of infection of 1 (MOI = 1), were grown in serum-free medium (SFM4MegaVir; HyClone, Logan, UT) for 4 days. Supernatant was clarified by centrifugation at 10,000 rpm for 30 min; virion particles in the supernatant were pelleted by a second centrifugation at 19,000 rpm for 16 hr. Viral pellets from the second centrifugation were resuspended in 1X phosphate buffered saline (PBS). These concentrated viruses were used to derive FIV.

An amount of 37% formaldehyde (Sigma-Aldrich, St. Louis, MO) was diluted with 1X PBS to 0.5% and adjusted to pH 7.2 with 10 N NaOH (Sigma-Aldrich, St. Louis, MO). The mixture was added to concentrated JEV viruses give a final formalin concentration of 0.05%. The formalin-treated virus (FIV) or untreated virus (untreated control virus; UCV) was incubated at 4°C for 49 days (the manufacture procedure for FICV provided by Adimmune Corporation in Taiwan), or at 22°C for 10 days [42]. FIV and UCV samples incubated at 4°C were collected every week and stored at -70°C for analysis. Nakayama virus specimens were inactivated by short-wavelength UV light at a distance of 3 cm on ice for 30 min or with a final concentration of 3% H_2_O_2_ (Fisher Scientific), pH 7.2, at 22°C from 2 to 8 hr, then stored at -70°C. The residual infectious viral titers of FIV, UCV or UV- or H_2_O_2_-treated viruses were assessed by micro-plaque assay.

### Antigen-capture (Ag) enzyme-linked immunosorbent assay (ELISA)

Antigen-capture ELISA (Ag-ELISA), described previously [48], was used to estimate E proteins concentrations in samples with anti-JEV mouse hyper-immune acitic fluid (MHIAF) (immunized with purified and live JEV) and determine the binding activity of MAbs. Briefly, a 96-well plate (Sigma-Aldrich, St. Louis, MO) was coated with rabbit anti-JEV polyclone (generated from rabbit immunized with pVAX-JEi VLP-expressing plasmid [49,50], and obtained from Dr. Chang GJ of US CDC, Fort Collins, CO) at 37°C for 1 hr, blocked with StartBlock blocking buffer (Pierce, Rockford, Ill.), then antigen was added at 40 ng per well for incubation at 4°C overnight. Antigen was incubated with MAbs and MHIAF, diluted with 5% skim milk, at 37°C for 1 hr, then peroxidase-conjugated goat anti-mouse IgG (H+L) (Jackson ImmunoResearch, West Grove, PA) at 37°C for 1 hr. Finally, 3,3’,5,5’-tetramethylbenzidine substrate (TMB; Neogen Corp., Lexington, KY) was added at 100 μl per well for the color reaction and reactions were stopped with 2N H_2_SO_4_ added at 50 μl per well; the OD_450_ values were recorded. The antigen concentration of UCV and FIV was estimated by the OD_450_ of the MHIAF. The MAb binding activities for UCV or FIV were determined by percentage reactivity estimated by the OD_450_ of UCV or FIV at the time relative to that at 0-day, respectively. All binding activities were adjusted by fold difference of antigen concentration, estimated by the OD450 of MHIAF against UCV or FIV at the time relative to that at 0-day, respectively and shown as mean±SD of two duplicates of two independent assays.

### SDS-PAGE and western blot analysis

JEVs and JEV virus-like particles (VLPs) were mixed with 5X SDS non-reducing sample buffer (315 mM Tris, pH 6.8, 50% glycerol, 5% SDS, 0.025% bromophenol blue), then loaded onto a 10% SDS gel. After separation, proteins were transferred to a nitrocellulose membrane, which was blocked with 5% skim milk. The proteins on the membrane were detected by use of mouse anti-JEV polyclonal antibody, MAbs or MHIAF and visualized by incubation with peroxidase-conjugated goat anti-mouse IgG (H+L) (Jackson ImmunoResearch, West Grove, PA); bands were developed by use of the LumiGOLD ECL Western Blotting Detection Kit (SignaGen Laboratories, Gaithersburg, MD). The intensities of bands were calculated by use of ImageJ version 1.44 (NIH, Bethesda, MD). The binding activity of anti-EDII 101/106/107 and anti-EDIII 329/331/389 antibodies against VLP was eliminated by introducing 101/106/107 and 329/331/389 mutations on VLP, respectively.

### Epitope mapping with VLPs

JEV VLPs were produced with the pVAX-JEi plasmid derived from the pCBJE plasmid [50], which encodes the prM and E protein regions of the SA14 strain genome. This plasmid was also used as the template for introducing mutations into the E protein by use of a site-directed mutagenesis kit (Stratagene, La Jolla, CA) as described [50,51]. The pVAX-JEi 101/106/107, 306, 329, 331, 332 and 389 amino acid mutants were introduced by mutagenesis primers (S1 Table), according to the manufacturer’s protocols, and mutation was confirmed by sequencing. The JEV VLP-expressing plasmids were electroporated into COS-1 cells by use of a 0.4-cm–electrode-gap cuvette and a Bio-Rad Gene Pulser II (Bio-Rad Laboratories, Hercules, CA) at 250 V and 975 μF; electroporated cells were recovered overnight at 37°C and incubated at 28°C to enhance VLP secretion. The secreted VLPs were analyzed by Ag-ELISA and used to evaluate the presence of epitope-specific antibodies.

### Focus-reduction micro-neutralization test (FRμNT)

To measure the neutralizing activity of the MAbs or in serum samples, briefly, 2.48×10^4^ Vero cells were added into 96-well plates for 24 hr at 37°C with 5% CO_2_. MAbs in pre-attachment assay or serum samples were inactivated at 56°C for 30 min, diluted in a two-fold series, mixed with 100 pfu JEV Nakayama strain for 1 hr, then shaken every 20 min. Monolayers of Vero cells were infected with the virus-antibody mixture for 1 hr at 37°C with 5% CO_2_; in contrast, in post-attachment assay, virus was bound on monolayers of Vero cells at 4°C for 1 hour, then incubated with a two-fold series diluted and inactivated MAbs at 4°C for 1 hour, and then was shift into 37°C incubator with 5% CO2 for 1 hour. After incubation, 1% methyl cellulose in DMEM containing 2% FBS was added to the 96-well plates for incubation for 36 hr at 37°C with 5% CO_2_, then plates were washed with PBS, fixed with 75% acetone, and air-dried in a hood. The fixed cells were stained with anti-JEV MHIAF for 40 min at 37°C. After a washing, peroxidase-conjugated goat anti-mouse IgG (Jackson ImmunoResearch, West Grove, PA) was added for 40 min, and virus-infected foci were identified by use of a Vector-VIP peroxidase substrate kit SK-4600 (Vector Laboratories, Burlingame, CA). The foci were counted manually under microscopy and used to calculate a sigmoidal dose-response for the focus reduction micro-neutralization test (FRμNT_50_) titers with use of GraphPad Prism v5.01.

### Mouse immunization and epitope-specific antibody response

Groups of 6-week old BALB/c mice (n = 5 mice per group) were vaccinated with three doses of Freund’s incomplete adjuvanted UCV-Nakayama, FIV-Nakayama or FICV. The first booster vaccination was given 2- week after the primary immunization and followed by a final booster at 4 weeks after the first booster vaccination. Serum samples were collected 2 weeks after the final booster vaccination.

An IgG antibody-capture ELISA (GAC-ELISA) described previously [52] was used to determine the titer of the epitope-specific antibodies in immunized mouse serum. Briefly, goat anti-mouse IgG (H+L) (KPL, Gaithersburg, MD) was coated on 96-well plates at 37°C for 1 hr, then plates were blocked with StartBlock blocking buffer (Pierce, Rockford, Ill.). Serum samples were serially diluted with wash buffer and added to plates at 37°C for 90 min. After a washing, 40 ng of the JEV antigens, wild-type (WT) or mutant JEV VLPs was added and mixtures were incubated at 4°C overnight. The IgG-capture antigens were detected by use of rabbit anti-JEV polyclonal antibody and peroxidase-conjugated goat anti-rabbit IgG (H+L) (Jackson ImmunoResearch, West Grove, PA) to detect antigen-bound rabbit anti-JEV polyclonal antibodies. The above steps were described for the Ag-ELISA. Total anti-immunogen IgG antibody was determined as endpoint titer. The epitope-specific antibody response was determined by the decreased reactivity titer against mutant VLP compared to WT VLP and was calculated as endpoint titer of (WT—mutant VLP).

The epitope-specific neutralizing antibody activity was determined as follows. The pVAX-JEi WT and EDIII 329/331/389 mutant plasmids were electroporated into COS-1 cells, cultured for 24 hr, and cells were resuspended in PBS. Diluted mouse serum samples were mixed with 10^7^ transformed COS-1 cells at 37°C on a shaker for 2 hr. Then the transformed COS-1 cells were removed by centrifugation at 3,000 rpm for 10 min and the supernatant containing unbound antibodies was serially diluted and mixed with 100 plaque-forming units (pfu) of JEV Nakayama strain at 37°C for 1 hr. The neutralizing activity was determined by FRμNT assay as described previously and neutralizing activity (%) calculated by [1-(plaque numbers of serum mixed with virus/plaque numbers of virus-only control)]* 100. The curves showing the neutralizing antibody activity at different dilutions were fitted by non-linear regression in GraphPad. The percentage of neutralizing antibodies that recognized the JEV EDIII 329/331/389 epitope was calculated by plaque numbers of serum post-adsorbed with [(WT VLP—EDIII 329/331/389 mutated VLP)/ (WT VLP—COS-1 cells)]* 100.

### Indirect immune-fluorescence assay (IFA)

The pVAX-JEi WT and EDIII 329/331/389 mutant plasmid-transformed COS-1 cells were seeded into wells of a chamber slide (Millipore, Billerica, MA), and cultured at 37°C overnight, then wells were fixed with 4% paraformaldehyde (Sigma, St. Louis, MO, USA) in PBS at room temperature for 20 min and washed with PBS. The fixed cells were made permeable by treatment with 0.1% Triton X-100 at 4°C for 5 min and washed with PBS, then wells were blocked with 3% bovine serum albumin (Sigma, St. Louis, MO, USA) in PBS at 37°C for 1 hr. Wells were stained with anti-JEV MHIAF and reacted with FITC-conjugated goat anti-mouse IgG (KPL, Gaithersburg, MD) in 1% Evans blue. Images were viewed under an OLYMPUS CKX41 microscope.

### Statistical analysis

Data are presented as mean±SD from two repeated experiments. Two-tailed Student’s *t* test was used for all analyses, and statistical significance was set at *p* <0.05.

## Results

### MAb binding activity of FICV

Most of the antibodies elicited by JEV infection or immunization are conformation-dependent and, for the most part, recognize the viral E protein and are able to help prevent virus infection [15,53]. Formalin inactivation of several human vaccines has been shown to result in antigenic alteration to the viral particles, which can be measured by the binding activity of specific MAbs [38,40], but the effect of formalin inactivation on commercially available JEV vaccines has not been evaluated. Previously, an established Ag-ELISA protocol was successfully used to determine the antigenic structure of JEV using a panel of anti-E protein MAbs [49,53].

**Fig 1:**
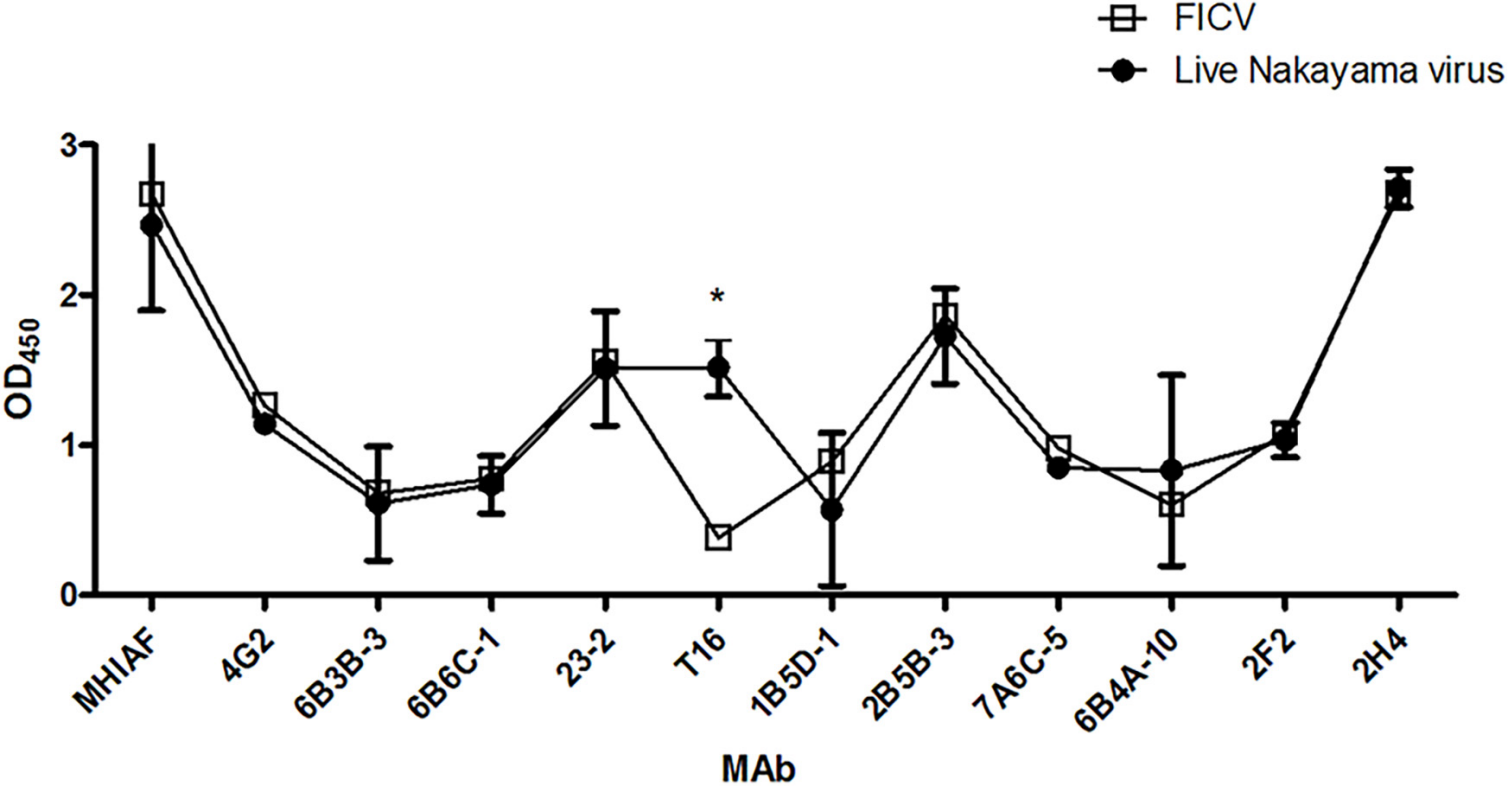
Monoclonal antibody (MAb) binding activity of formalin-inactivated commercial JEV vaccine (FICV) and live Nakayama strain determined by Ag-ELISA. Three types MAbs were used: group cross-reactive (4G2, 6B6C-1, 6B3B-3 and 23–2), JEV serocomplex cross-reactive (T16, 1B5D-1, 2B5B-3, 7A6C-5, and 6B4A-10) and JEV type-specific (2F2 and 2H4). Data are OD_450_ of mean±SD from two duplicates, and the significant difference was indicated as an asterisk (*p*<0.05).

First, we evaluated the antigenic differences between the live Nakayama virus and FICV by Ag-ELISA using a panel of eleven anti-flavivirus E-protein MAbs [45–47]. The same antigen concentration (estimated by Ag-ELISA using JEV-specific MHIAF) of live virus and FICV was used throughout the experiments. Live Nakayama virus and FICV showed similar binding pattern for ten of the eleven tested MAbs, with the exception being T16 MAb (Fig 1). The binding activity with T16 MAb was significantly lower for FICV than for the live Nakayama virus (*p*<0.05) with end-point titers of 10^5.39^ and 10^3.48^ for the live Nakayama virus and FICV respectively. The MAb binding pattern suggests that the antigenic structure of FICV differs from that of the live Nakayama virus.

### MAb binding activity of formalin-inactivated JEVs (FIV-JEVs)

**Fig 2:**
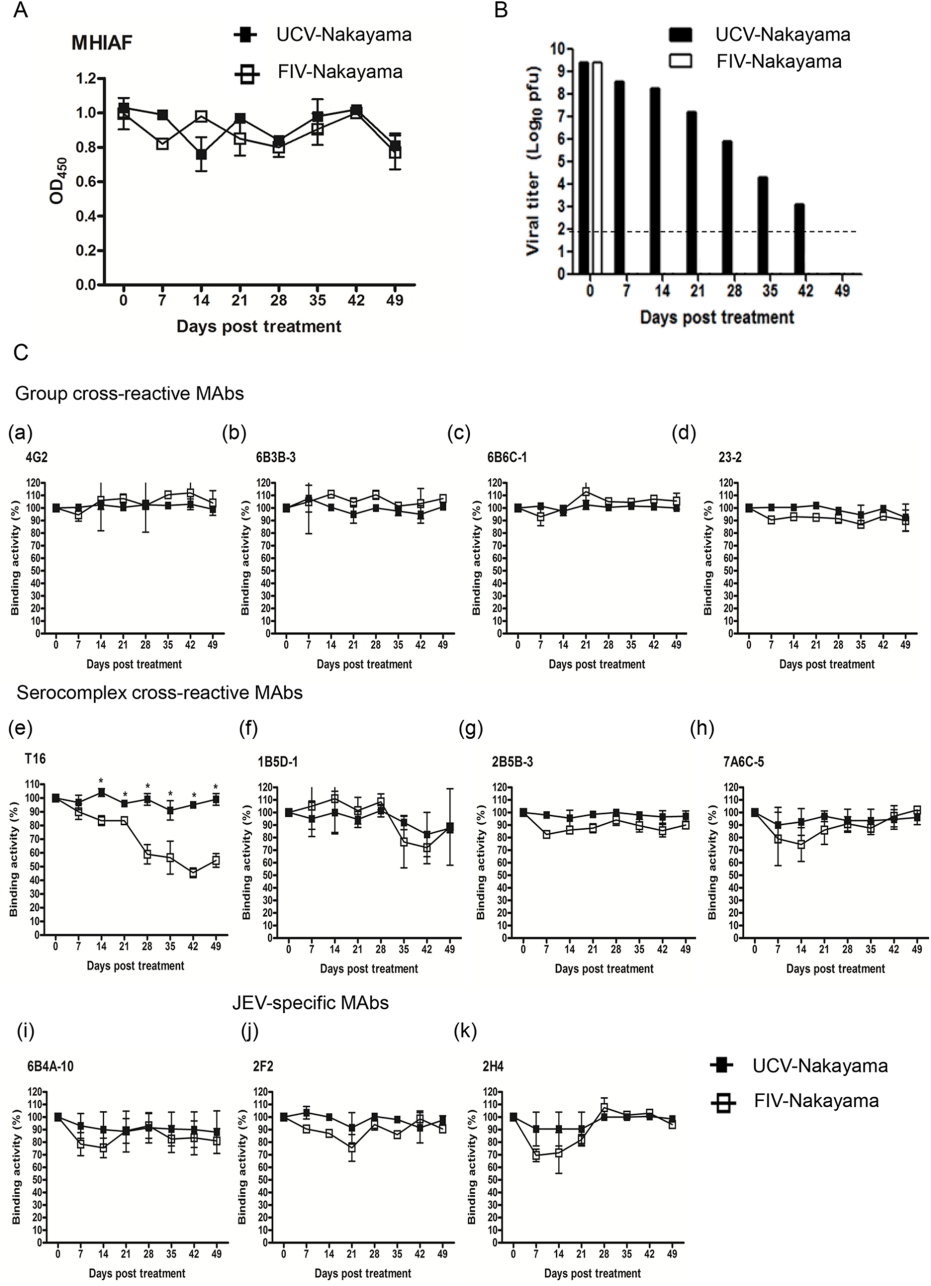
Characteristics of untreated control Nakayama virus (UCV-Nakayama) and formalin-inactivated Nakayama virus (FIV-Nakayama) at 4°C for 49 days. (A) Antigenic reactivities of UCV- and FIV-Nakayama were monitored by Ag-ELISA using MHIAF. Data are OD_450_ of mean±SD from two duplicates. (B) Infectious viruses were determined by plaque assay after formalin treatment. (C) MAbs binding activity of UCV-Nakayama and FIV-Nakayama were evaluated by Ag-ELISA. The binding activities were adjusted to antigen concentration according to the OD_450_ of MHIAF, compared to day 0 (as 100%). Data are mean±SD from two duplicates, and the significant difference was indicated as an asterisk (p<0.05).

The decrease in binding activity of T16 MAb against FICV might be due to procedure variation during vaccine manufacture, which include differences in the formalin inactivation, differences in the virus purification process, changes in the sub-strains used and differences in the passage history of the Nakayama virus used between the vaccine production virus strain and the live Nakayama virus used in this experiment. To rule out the potential influence of sub-strain differences on the E structure and focus on the effect of formalin treatment on antigenic modification of Nakayama virus, we subjected laboratory-grown concentrated Nakayama virus to either formalin inactivation (FIV-Nakayama) or without formalin at 4°C for 49 days (untreated control virus-Nakayama virus, UCV-Nakayama). The antigenic reactivity of FIV-Nakayama and UCV-Nakayama, as determined by Ag-capture ELISA with anti-JEV MHIAF, remained constant (Fig 2A). The infectivity of FIV-Nakayama decreased drastically to below the detection limitation after 7 days treatment under these conditions; however, the infectivity of UCV-Nakayama decreased gradually over time and only became undetectable after 49 days (Fig 2B).

Ten of the eleven MAbs, with the exception of T16, showed similar binding activity with FIV-Nakayama and UCV-Nakayama preparations collected at most time points by Ag-ELISA (Fig 2C). The binding activity of the JEV serocomplex cross-reactive T16 MAb against FIV-Nakayama was significantly decreased at the 14-day collection point (14-DC) with this sample having only 83% of the binding activity of the 0-DC sample. This decrease in binding of T16 against FIV-Nakayama was time-dependent; with only 55% binding activity remaining at 49-DC (Fig 2C, panel e). Unlike T16, 2B5B-3 and 2F2 binding against FIV-Nakayama declined at an early time point compared to UCV-Nakayama, but this was not observed at later time points. This result is consistent with the observation that only T16 exhibiting a decreased binding activity against FICV by Ag-ELISA (Fig 1). Therefore, we believed that the decreased binding activity of T16 MAb against FICV is the result of formalin inactivation and is not related to potential antigenic differences related to the sub-strain of virus or associated with the passage history of virus.

**Fig 3:**
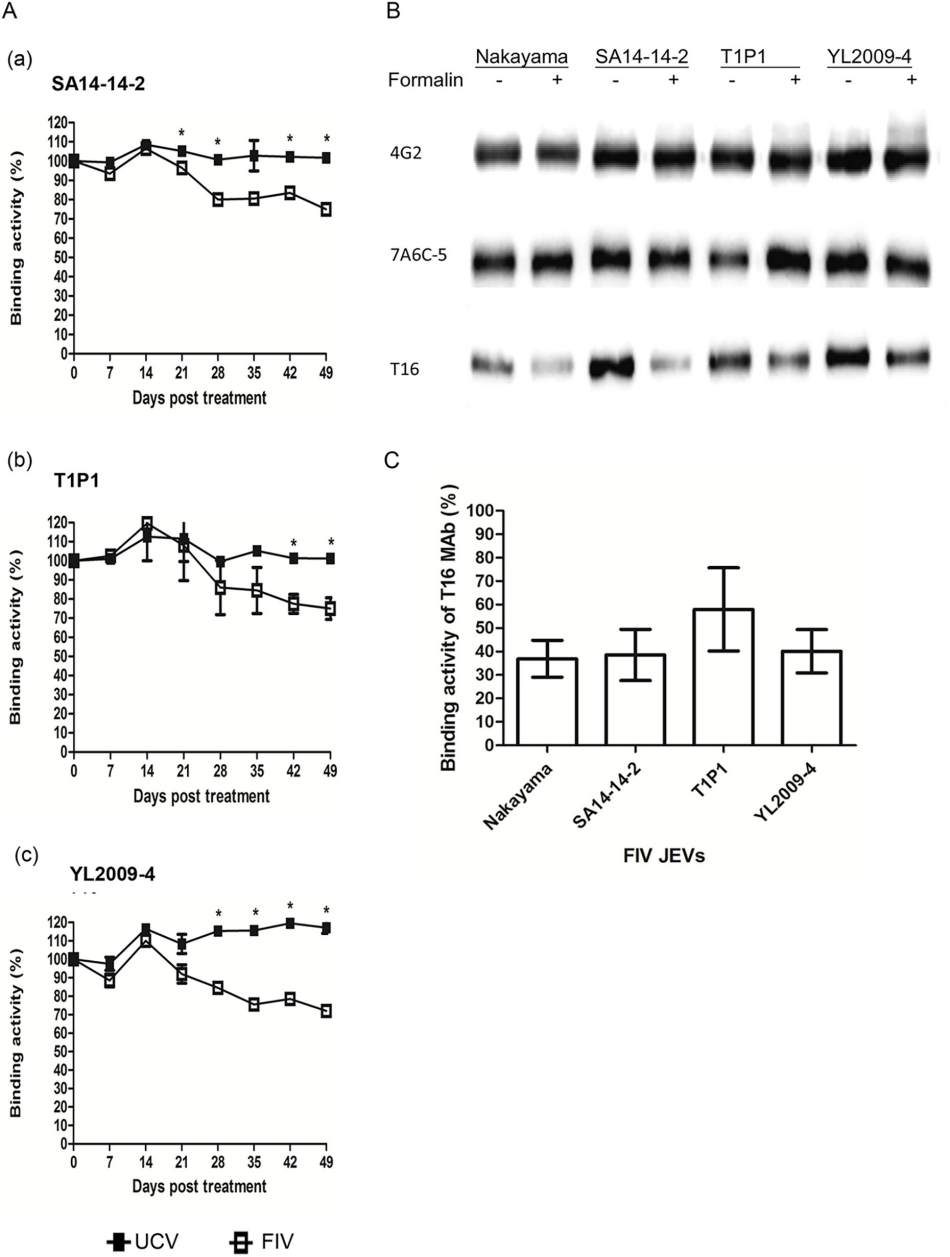
T16 MAb binding activity of formalin-inactivated virus (FIV) JEVs. (A) Ag-ELISA of binding activity of T16 MAb with JEV genotype III SA14-14-2 (vaccine strain), T1P1 (naturally attenuated strain), and genotype I YL2009-1 (wild-type strain) inactivated with 0.05% formalin at 4°C for 49 days. Data are mean±SD from two duplicates, and the significant difference was indicated as an asterisk (*p*<0.05). (B) Western blot analysis of the viral envelope protein on FIV and UCV JEVs detected with T16, 4G2 and 7A6C-5 MAbs and (C) quantification of T16 MAb binding activity with E of the UCV JEVs by the value of UCV JEVs shown as 100% of the FIV JEVs value. Data are mean±SD from two duplicates, and the significant difference was indicated as an asterisk (*p*<0.05).

To rule out formalin-induced antigenic modification occurring at strain-specific amino acids [36,54], we prepared three different strains of JEV, the SA14-14-2 GIII vaccine-strain virus, the T1P1 naturally attenuated GIII virus and the YL2009-4 GI virus [44], and then applied the same formalin inactivation procedures to all three viruses; this was followed by measurement of their MAb binding by Ag-ELISA using a subset of eight MAbs. The pattern of MAb binding activity obtained with these viruses was similar to that obtained with the Nakayama virus with or without formalin treatment (Fig 3 and S1 Fig). Again, at 49-DC, the binding activity of T16 MAb was significantly decreased to 75%, 75%, and 72% for FIV-SA14-14-2, FIV-T1P1, and FIV-YL2009-4, respectively (Fig 3A). To further confirm that the decrease in binding activity of T16 MAb against the E protein was due to formalin inactivation, these viral preparations with or without formalin treatment were analyzed by non-reducing SDS PAGE followed by Western blotting using the T16. 4G2 and 7A6C-5 MAbs, which have similar Ag-ELISA binding activity against the FIV-JEV and the UCV-JEV antigens, were included for comparison (Figs 2C and 3 and S1 Fig). The intensity of the E protein band, when detected by 4G2 and 7A6C-5 of the various FIV-JEV and UCV-JEVs, including Nakayama, SA14-14-2, T1P1, and YL2009-4, were similar; however, the intensity detected by T16 was lower against the FIV-JEVs than against the UCV-JEVs (Fig 3B). By way of comparison, at 49-DC, the formalin-treated Nakayama, SA14-14-2, T1P1, and YL2009-4 viruses were found to have reduced T16 binding intensities of only 36%, 38%, 57%, and 40% of the UCV-JEVs, respectively (Fig 3C). Therefore, formalin inactivation, when it affects the antigenic structure of JEV E protein, would seem not to be viral strain-specific and is likely to occur at the virion level, perhaps affecting the E monomer containing disulfide bonds.

### Epitope mapping and neutralizing activity of T16 MAb

**Fig 4:**
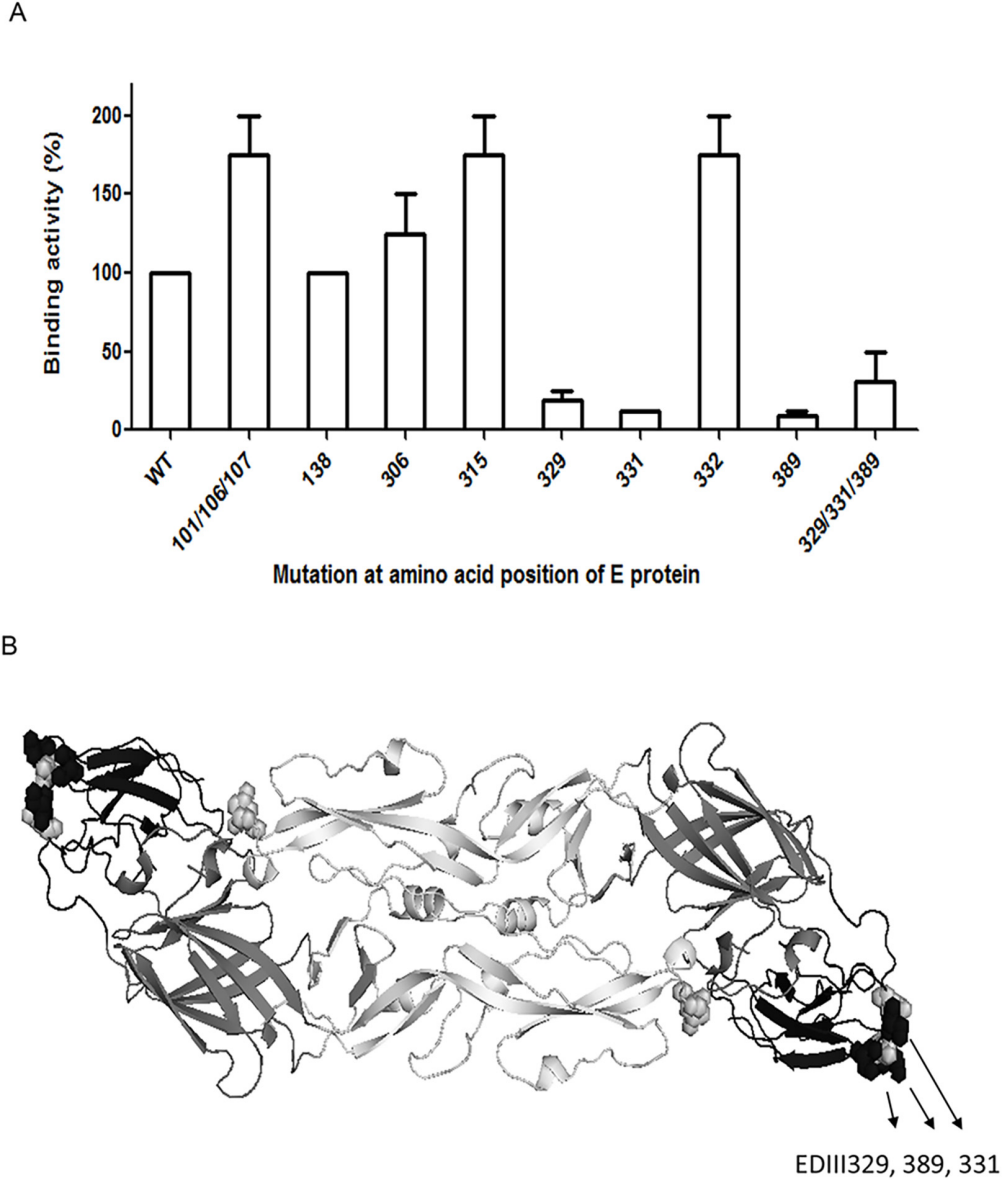
Epitope mapping of T16 MAb. (A) T16 MAb binding activity with mutation(s) introduced on the envelope (E) protein of JEV virus-like particles (VLPs) at amino acid residues 101, 106, 107, 306, 329, 331, 332 and 389 was determined by Ag-ELISA. Binding activity of T16 MAb against wild-type (WT) VLPs was set as 100%. (B) Stereoview of homologous E protein dimer; the amino acid residues affecting the binding activity of T16 MAb are in black.

To localize the formalin-modified epitope on E protein, we mapped the epitope recognized by T16 MAb. The antigenic structure of non-infectious JEV VLP is similar to that of the virion particle [50,51]. T16 MAb is a JEV serocomplex cross-reactive antibody and we previously found that amino acid residues 101, 104, and 106, which are present in EDII, and amino acid residues 315, 331 and 389, which are present in EDIII, are important for the binding of JEV serocomplex cross-reactive MAbs [49,53]. Thus we used a VLP-expressed plasmid to locate the formalin-modified epitope recognized by T16 MAb. JEV VLPs with EDIII amino acid substitutions S329A, S331K, and D389G, but not JEV VLPs with amino acid substitutions W101G/G106K/L107D, E138K, E306G, A315G and D332R, showed decreased binding to T16 MAb (Fig 4A). Amino acids 329 and 331 are located within the BC loop of the EDIII of JEV and amino acid 389 is located within the FG loop of the EDIII of JEV; these three amino acids are likely candidates to undergo modification during formalin inactivation (Fig 4B).

**Fig 5:**
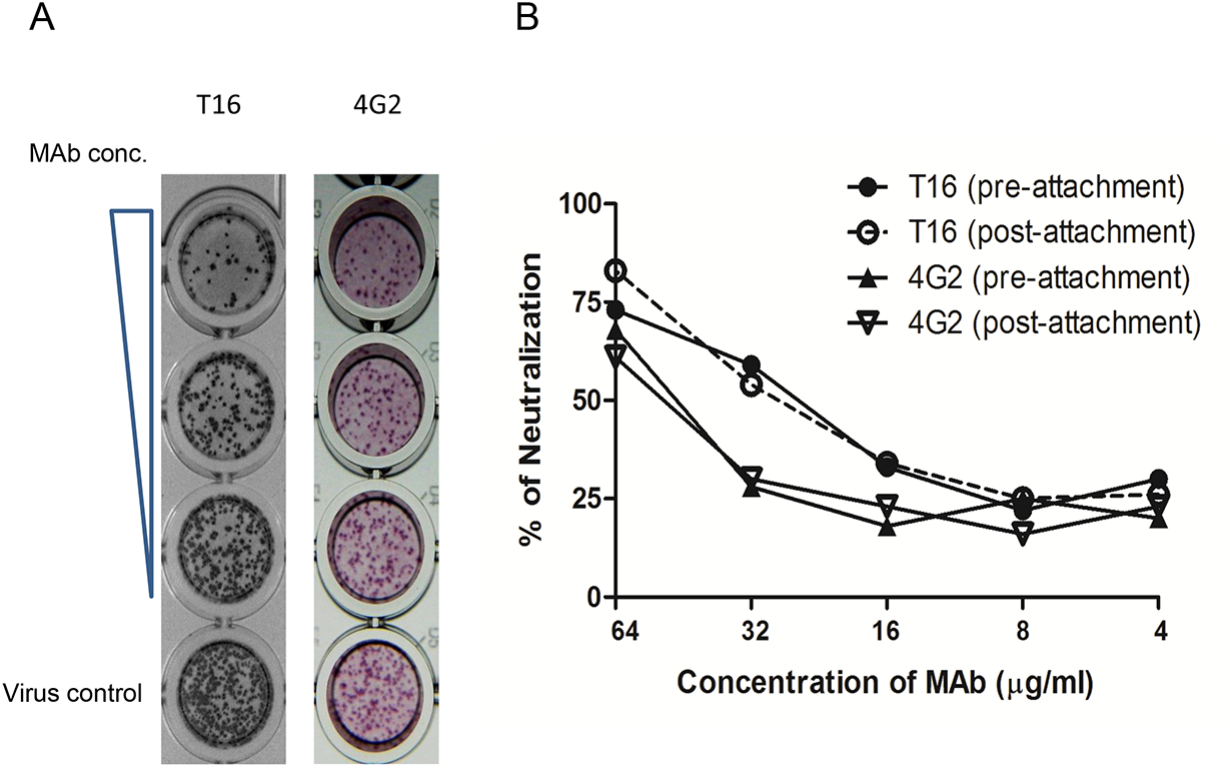
Neutralizing capacity of T16 MAb. (A) Neutralizing antibody titer of T16 was measured against T1P1 virus in Vero cells using a focus-reduction micro-neutralizing test (FRμNT), and (B) neutralizing mechanism of T16 MAb added before or after T1P1 virus binding to Vero cells. 4G2 MAb was used as a control for inhibiting viral fusion at the post-attachment step [58]. Data are mean±SD from two duplicates.

The BC loop of EDIII contains critical residues recognized by neutralizing MAbs against JEV and West Nile virus, while the FG loop of EDIII is involved in host tropism [55–57]. Therefore, we analyzed the neutralizing ability of T16 by FRμNT assay (Fig 5A). It was found that the FRμNT_50_ potency of T16 MAb was 21.8 μg/ml. 4G2 MAb neutralizes and inhibits flaviviral infection at the post-attachment step [58]. Thus, we used both 4G2 and T16 MAbs to determine the mechanism of viral neutralization. MAb was used to bind to JEV before infecting Vero cells in order to carry out a pre-attachment assay. Alternatively, MAb was added to JEV-bound cells in order to carry out a post-attachment assay. The neutralizing patterns of 4G2 and T16 MAbs were similar regardless of whether either MAb was added before or after viral attachment (Fig 5B), which indicates that T16 MAb seems to inhibit JEV at a post-attachment step.

### Antibodies against the EDIII 329/331/389 epitope in vaccinated mice

**Fig 6:**
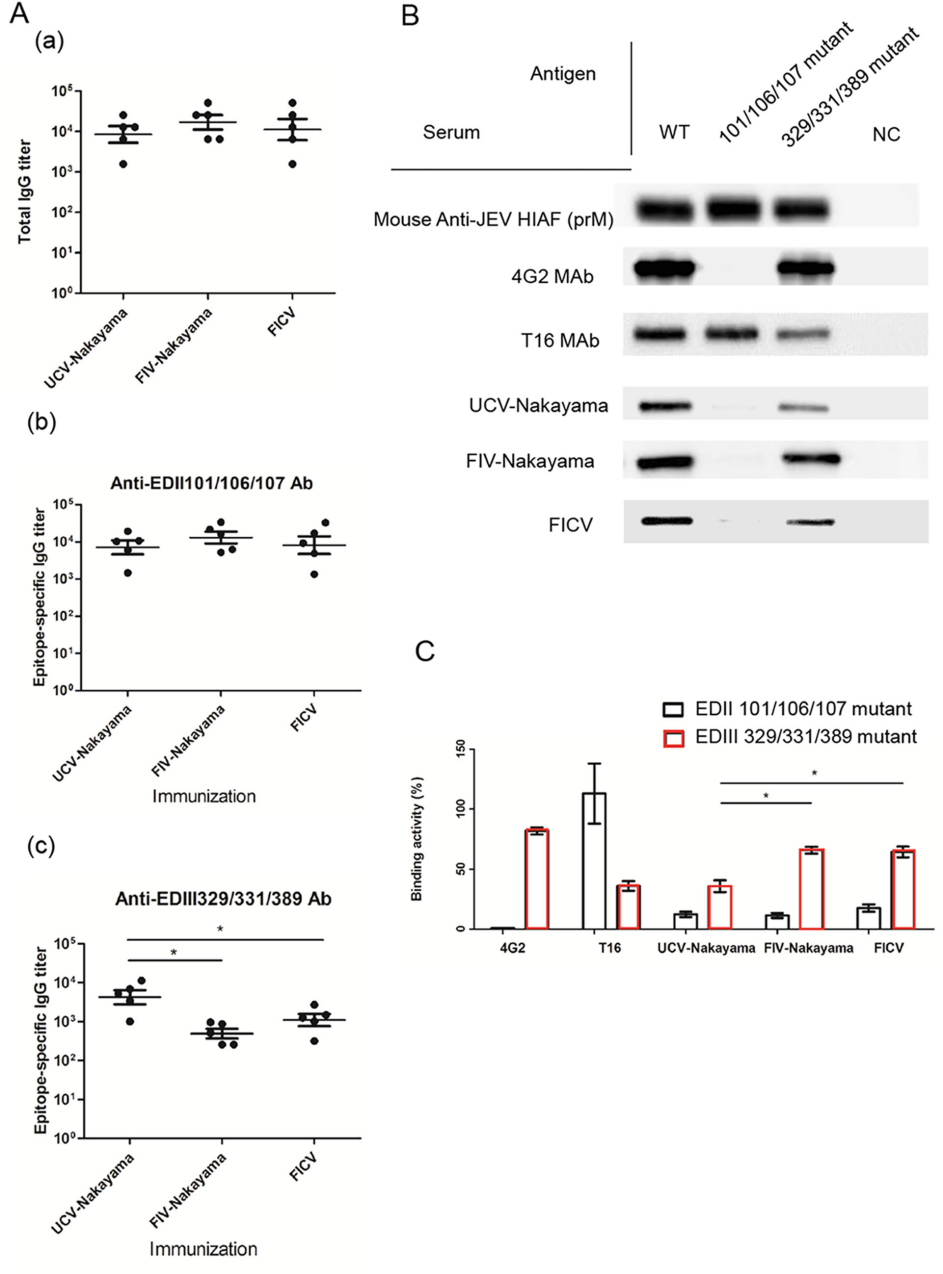
The immunogenicity of JEV EDIII 329/331/389 epitope on viral E protein of FIV-Nakayama in mice. (A) Serum specimens were collected from mice vaccinated with UCV-Nakayama, FIV-Nakayama, and FICV (n = 5 mice in each group). Each point represents one serum sample. Horizontal bars are mean and whiskers are SD. The total IgG antibody response was measured by IgG antibody-capture (GAC)-ELISA (A-a) and the epitope-specific antibody response of mouse serum on EDII 101/106/107 (A-b) and EDIII 329/331/389 (A-c) of E protein. The significant difference was indicated as an asterisk (*p*<0.05). (B) Western blot analysis of the viral premembrane (prM) or E protein of JEV wild-type (WT), EDII 101/106/107 mutant and EDIII 329/331/389 mutant virus-like particles (VLPs) detected by mouse anti-JEV HIAF, 4G2, T16, and UCV-Nakayama–, FIV-Nakayama–and FIVC-immunized mouse serum and (C) quantification with the binding activity of E protein of WT VLP set to 100%. Data are mean±SD from two duplicates, and the significant difference was indicated as an asterisk (*p*<0.05). NC, COS-1 cell lysates.

To evaluate the influence of the T16 epitope (EDIII 329/331/389) on the immunogenicity of formalin-treated JEV antigens, we further investigated IgG antibody responses and the properties of antibodies against EDIII 329/331/389 in vaccinated mice. Female BALB/c mice were vaccinated with UCV-Nakayama, FIV-Nakayama, or FICV and then post-vaccination serum samples were analyzed by the IgG-capture ELISA using wild-type, EDIII 329/331/389-mutated and EDII 101/106/107-mutated VLPs. The EDII 101/106/107-mutated VLPs eliminate the immunodominant B-cell epitope, conserved in all flaviviruses as well as inducing cross-reactive, non-neutralizing and/or low-neutralizing antibodies [49,59]. The total JEV-specific IgG elicited by all three immunogens were similar (*p*>0.05) with the average titer end-points being 8.5×10^3^, 1.5×10^4^, and 1.1×10^4^ for UCV-Nakayama, FIV-Nakayama, and FICV-immunized mice, respectively (Fig 6A, panel a). We determined the antibody responses that recognized the EDII 101/106/107 epitope and the EDIII 329/331/389 epitope by calculation the decreased reactivity titer against EDII 101/106/107-mutated and EDIII 329/331/389-mutaed VLP compared to the wild-type VLP. The titer of antibodies recognizing the EDII 101/106/107 epitope was similar (*p*>0.05) for all serum from mice vaccinated with UCV-Nakayama (10^3.9^, range 10^3.2^–10^4.3^), FIV-Nakayama (10^4.1^, range 10^3.7^–10^4.5^), and FICV (10^3.9^, range 10^3.1^–10^4.5^) (Fig 6A, panel b). In contrast, the titer of EDIII 329/331/389 epitope-specific antibodies was significantly lower (*p*<0.05) for serum from mice vaccinated with FIV-Nakayama (10^2.7^, range 10^2.4^–10^3^) and FICV (10^3^, range 10^2.5^–10^3.4^) compared to UCV-Nakayama (10^3.6^, range 10^3^−10^4.1^) (Fig 6A, panel c).

Based on the above, we suspected that FICV-immunized children might produce a similarly lower proportion of EDIII 329/331/389 epitope-specific antibody. Twelve FICV-immunized children serum samples were found to show a lower level for the EDIII 329/331/389 epitope-specific IgG antibodies, namely 23% (10–46%) (S2 Table); this result closely resembles the antibody reactions elicited in the FIV-Nakayama–immunized and FICV-immunized mice.

To confirm the results obtained by epitope-specific IgG ELISA, the FIV-Nakayama and FICV immunized mouse serum samples were examined by Western blot analysis using the same concentration of WT, EDII 101/106/107-mutated VLP and EDIII 329/331/389-mutated VLP (Fig 6B) and the results quantified against standardized protein concentrations (Fig 6C). The prM protein of all of the JEV VLPs, including the WT antigen, the EDII 101/106/107-mutated antigen and the EDIII 329/331/389-mutated antigen, were equally recognized by the anti-JEV MHIAF. Furthermore, the EDII 101/106/107-mutated VLP and EDIII 329/331/389-mutated VLP could not be recognized or showed significantly decreased recognition with the 4G2 and T16 MAbs, namely <1% and 36% reactivity, respectively. The serum collected from mice vaccinated with UCV-Nakayama was less able to bind to the EDIII 329/331/389-mutated VLP (35%) than FIV-Nakayama and FICV (65% and 64%, respectively), but this was not true for the EDII 101/106/107-mutated VLP (12%, 11%, and 17%, respectively) (Fig 6B and 6C). The results of the epitope-specific IgG ELISA and Western blot analysis are consistent and indicate a stronger immunogenicity of the EDIII 329/331/389 epitope on UCV-Nakayama than that on FIV-Nakayama and FICV. Therefore, formalin-inactivated Nakayama virus or vaccine in immunized mice was only able to affect the induction of antibodies recognizing the EDIII 329/331/389, but was not able to affect the induction of antibodies recognizing the EDII 101/106/107 epitope.

### Neutralizing activity of the EDIII 329/331/389-specific antibodies

The protective efficacy of vaccines against JEV infection is positively associated with the presence of neutralizing antibodies in mice [60]. Based on this we evaluated the correlation between the production of neutralizing antibodies binding to EDIII 329/331/389 across various vaccines. The contribution of EDIII 329/331/389-specific antibodies to the viral neutralizing activity was determined by FRμNT using mouse serum specimens elicited by UCV-Nakayama, FIV-Nakayama or FICV. Serum samples were pre-adsorbed with the same number of normal COS-1 cells (adsorption control), or COS-1 cells expressing WT or EDIII 329/331/389-mutated JEV VLPs. The level of VLP-expressing COS-1 cells was estimated by staining with anti-JEV MHIAF at 24 hr after transformation with the JEV WT or EDIII 329/331/389 mutant plasmid. The IFA positive rates were similar at about 85% for COS-1 cells transformed with either of the plasmids (S2 Fig).

**Fig 7:**
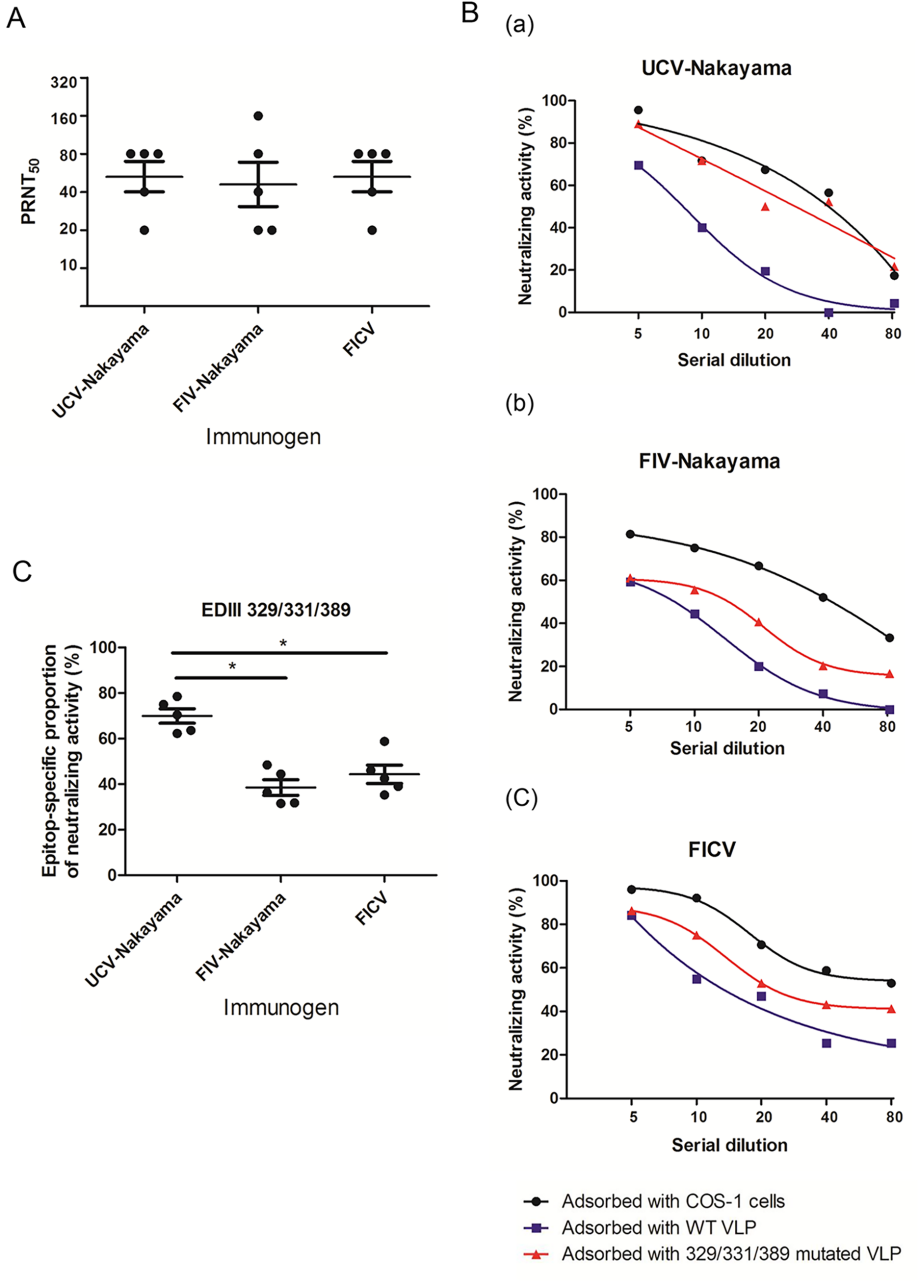
Neutralizing capacity of immunized mouse serum and antibody recognizing JEV EDIII 329/331/389 epitope in immunized mouse serum. (A) Focus-reduction micro-neutralizing test (FRμNT) of UCV-Nakayama–, FIV-Nakayama–and FICV-immunized serum (n = 5 mice in each group). Each point represents one serum sample. Horizontal bars are mean and whiskers are SD. (B) The neutralizing activity of serum samples determined after adsorption with COS-1 cells (black line) and VLP-expressing COS-1 cells including WT VLP (blue line) and EDIII 329/331/389 VLP (red line). The neutralizing activity (%) was presented and calculated by [1-plaque numbers of (serum mixed with virus/ virus-only control)]* 100. The curves of neutralizing antibody at different dilution were fit by non-linear regression with GraphPad. These representative data were from a single serum sample for each type. (C) The percentage of neutralizing antibodies recognizing JEV EDIII 329/331/389 epitope was calculated with plaque number of serum post-adsorbed with [(WT VLP- EDIII 329/331/389 mutated VLP)/(WT VLP-COS-1 cells)]*100 in FRμNT assay. Each point represents one serum sample. Horizontal bars are mean and whiskers are SD. The significant difference was indicated as an asterisk (*p*<0.05).

The pre-adsorption neutralizing antibody titers of mouse serum immunized with UCV-Nakayama, FIV-Nakayama or FICV were similar, with FRμNT_50_ titers of 52 (20–80), 46 (20–160), and 52 (20–80), respectively (Fig 7A). We then measured the post-adsorption FRμNT_50_ titers (Fig 7B) to determine the contribution of the EDIII 329/331/389-specific antibodies to the viral neutralizing activity. The neutralizing antibody titers were lower for serum post-adsorbed with the WT JEV VLP-expressing COS-1 cells than for serum post-adsorbed with normal COS-1 cells using serum samples elicited by all three vaccines (Fig 7B). However, the post-adsorption serum specimens using JEV EDIII 329/331/389-mutant VLP-expressing COS-1 cells showed a significant reduction in their neutralizing antibody titers activity when serum from either FIV-Nakayama-immunized mice or FICV-immunized mice was used, but not when the serum from UCV-Nakayama–immunized mice was used.

The differences in neutralizing activity of the serum samples after adsorption with COS-1 cells expressing the WT VLP or EDIII 329/331/389-mutant VLP may have been due to the contribution made by EDIII 329/331/389-specific antibodies. When the results were fitted using non-linear regression analysis (Fig 7C), this showed that the contribution of EDIII 329/331/389-specific antibodies to neutralizing antibody activity was proportionally higher (69%, range 62–78%) using the serum from UCV-Nakayama-immunized mice than when the serum from FIV-Nakayama-immunized mice (38%, range 31–48%) or FICV-immunized mouse serum (44%, range 35–58%) was used. Thus, formalin modification of the EDIII 329/331/389 epitope would seem to affect the production of neutralizing antibodies.

### A search for alternative methods to be used for the production of inactivated JEV vaccine

**Fig 8:**
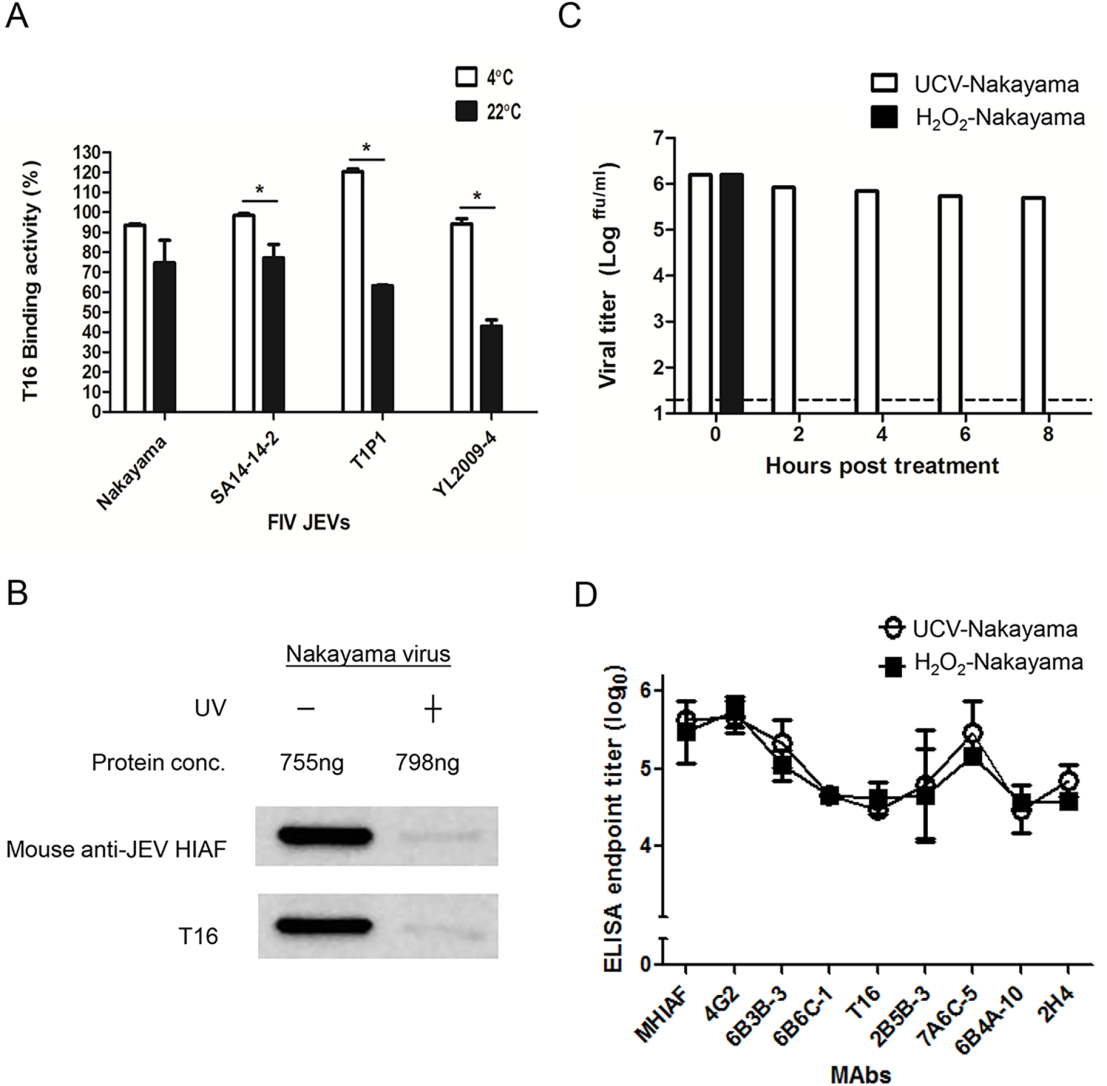
T16 MAb binding activity of JEVs inactivated at different temperature or with UV and H_2_O_2_. (A) The binding activity of FIV JEVs was determined by Ag-ELISA and adjusted to antigen concentration by OD_450_ of MHIAF, compared to day 0 (as 100%). Data are mean±SD of two duplicates, and the significant difference was indicated as an asterisk (*p*<0.05). (B) Western blot analysis of viral E protein with similar protein concentration of UV-treated and -untreated Nakayama virus detected with mouse anti-JEV HIAF and T16. (C) Plaque-forming assay of UCV-Nakayama virus titers after H_2_O_2_ treatment at 0, 2, 4, 6, 8 hr. (D) The binding activity of MAbs with UCV-Nakayama virus treated with H_2_O_2_ for 2 hr was shown as endpoint titers in Ag-ELISA. Data are mean±SD from two duplicates.

A previous report has suggested that JEV inactivation by formalin at 22°C for 10 days might be more immunogenic than inactivation at 4°C for 49 days [42]. Therefore, we asked if inactivation temperature (4°C *vs*. 22°C) and inactivation duration (49 days *vs*. 10 days, respectively) is able to influence T16 modification. We measured the T16 MAb binding activity of the JE Nakayama, SA14-14-2, T1P1, and YL2009-4 viruses treated with formalin at 4°C or 22°C for 10 days (Fig 8A). At 10-DC, the T16 MAb binding activity against FIV-Nakayama, FIV-SA14-14-2, FIV-T1P1, and FIV-YL2009-4 were all lower at 75%, 77%, 63%, and 43% at 22°C than at 4°C, where the results were 94%, 98%, 120%, and 94%, respectively. Thus T16 epitope modification is present on FIV-JEVs treated either at 22°C for 10 days (remaining 43–77% of T16 MAb binding activity) or at 4°C for 49 days (remaining 55–75% of T16 MAb binding activity) (Figs 2C and 3A).

UV has also been used to inactivate viruses in the past and such UV-inactivated viruses are able to induce protective humoral immunity [61,62]. Surprisingly, UV-inactivated Nakayama virus only weakly bound anti-JEV MHIAF and T16 MAb when assessed by Western blot analysis, which indicates that the antigenic structure of Nakayama virus might be severely altered by UV irradiation (Fig 8B).

Hydrogen peroxide (H_2_O_2_) can be used as a biocide and is known to interact with amino or sulfhydryl groups on antigens. Amanna *et al*. recently reported that H_2_O_2_-inactivated viruses are still able to induce protective cellular and humoral immunity [63,64]. Therefore, we followed their protocol and inactivated the Nakayama virus with 3% H_2_O_2_ at 22°C from 2 to 8 hr. Two-hours of H_2_O_2_ treatment reduced viral infectivity by at least 42000-fold to under the detection limitation (Fig 8C). Ag-capture ELISA revealed that the binding activities of the T16 and other cross-reactive MAbs against the UCV-Nakayama and the H_2_O_2_-treated Nakayama virus were the same after 2-hr of treatment at 22°C. This suggests the antigenic structure of the Nakayama virus remained intact after H_2_O_2_ inactivation (Fig 8D).

## Discussion

Several countries, including Japan, South Korea, and Taiwan, have successfully reduced the number of JE clinical cases by using inactivated JEV vaccines, but more effective and safe alternative vaccines are still needed [65–67]. The factors that affect the effectiveness and safety of JEV vaccines are the virus strain, method for viral cultivation, vaccine purity and vaccine formulation [68]; however, the effect of formalin inactivation on the quality of vaccine has never been studied. This is important because formalin-induced hypersensitivity has been found associated with risk of enhanced disease during subsequent infection with respiratory syncytial virus (RSV), and formalin inactivation altered the antigenicity of poliovirus [38,41,69]. Antigenic characterization of formalin-inactivated poliovirus vaccine by using a panel of MAbs revealed that modification of antigenicity is time-dependent [38]. Using a previously collected panel of anti-flavivirus MAbs and the established Ag-ELISA [49,53], we found that only the T16 MAb binding domain was time- and temperature-dependently altered by formalin inactivation. This observation suggested it might be valuable to evaluate the effect of residual formalin in formulated bulk on vaccine shelf life in the future. Importantly, regardless of the JEV strain used, formalin treatment altered the T16 epitope of all tested JE viruses. In contrast, epitopes recognized by 2B5B-3 and 2F2 MAbs on FIV-Nakayama were temporarily modified for specimen collected at early time point. Modification of these two epitopes was Nakayama strain-specific and was reversible since this phenomenon was only observed in the early time point specimen of formalin-treated Nakayama alone.

Among anti-flavivirus antibodies, most of the virus-specific, non–cross-reactive, and EDIII-recognizing antibodies have strongly neutralizing activity, and most of the cross-reactive and EDII- or EDI-recognizing antibodies have weak or no neutralizing activity [16,70]. T16 MAb is a JEV-derived, JEV-serocomplex cross-reactive antibody. It shows weakly neutralizing activity at the post-attachment step *in vitro*. However, antibodies that comprise a large portion of the antibody response after WNV infection have only weak neutralizing activity *in vitro* but still provide therapeutic protection *in vivo* via the immune complement system [71]. The amino acid residues in both EDII and EDIII of the E protein are important to the binding of JEV serocomplex cross-reactive MAbs [49,53]. We determined the binding of T16 MAb to JEV VLPs by the amino acid positions EDII-104, -329, -331, and -389 but used only EDIII 329/331/389-mutated VLPs to analyze epitope-specific antibody responses because EDII-104–mutated VLPs showed reduced secretion. Interestingly, the T16 epitope overlaps with the JEV-specific highly neutralizing E3.3 MAb epitope [56]. This result provides additional support that most E-protein epitopes within flaviviruses are overlapping [53].

Formalin is known to mainly react with the amino and thiol groups of amino acids to form methylol groups, which is followed by the formation of Schiff-base adducts; this reaction is reversible. These Schiff-bases adducts can cross-link to functional groups of various amino acids, such as arginine, tyrosine, tryptophan, histidine, glutamine, lysine, and cysteine, forming non-reversible methylene bridges [35]. Thus, the epitope of T16 MAb, namely glycine 104, serine 329, serine 331 and aspartic acid 389, are likely not directly modified by formalin but are possibly influenced by nearby amino acids, including those at 105, 335, 336, 387, 390, and 391, and such cross-linking might directly or indirectly affect the conformational structure of the T16 epitope. Formalin treatment did not alter T16-overlapped epitopes recognized by 4G2 and 6B6C-1. T16 MAb might be more sensitive to this formalin-generating modification on the non-overlapped residue(s) essential for T16 recognition. Currently we still do not know residues specifically reacting with formalin.

Structural differences and amino acid variation in flavivirus immunogens, such as whether the virions are mature or immature, VLPs, or EDIII alone, may also affect the immunogenicity, antibody profile, and neutralizing potency elicited [72–75]. For example, EDIII-reacting antibodies show high neutralizing potency, but the recombinant EDIII immunogen induces low avidity and low titers of neutralizing antibodies against the virus [72]. In this study, we found that formalin inactivation altered the structure of the JEV E protein and thus affected the profile of induced antibodies. In this study, T16 epitope was the only epitope affected by the formalin inactivation; however, whether the T16 epitope is the only E structure alteration affecting the profile of antibodies elicited by formalin-inactivated vaccines/viruses is unknown because the T16 epitope, EDIII 329/331/389, was not directly reactive with formalin. The formalin-modified EDIII 329/331/389 region was found less immunogenic and had less of a contribution to the neutralizing activity, despite non-significant differences in neutralizing antibody titers among UCV-Nakayama–immunized mice and FIV-Nakayama–immunized mice.

Weak-neutralizing and non-neutralizing epitopes were located in the fusion peptide, and the introduction of mutations into the fusion peptides of the VLP disrupted the binding activity of anti-fusion loop MAbs. The fusion peptide mutant reduced the immunogenicity of the fusion peptide but retained its ability to evoke neutralizing antibodies [76,77]. Thus, the formalin-modified region affects the profile of vaccine-induced antibodies and alters the distribution of neutralizing antibodies. We did not determine the effect of formalin inactivation on the T-cell response, which needs to be addressed because a negative effect of formalin-inactivation on the influenza-virus T-cell response has been documented and T-cell immunity plays a role in how vaccines protect against JEV infection [37,78,79].

The use of epitope scaffolds or deglycosylation has successfully exposed immunorepressive and cryptic epitopes and enhanced immunogenicity in HIV or redirected the antibody response in simian immunodeficiency virus [80,81]. We found the titers of EDIII 329/331/389-reactive antibodies higher among UCV-Nakayama–than FIV-Nakayama–or FICV-immunized mice and use of EDII 101/106/107-reactive antibodies gave similar results. Previously, we found that EDII 101/106/107 and EDIII 329/331/389 form an overlapped epitope for flavivirus group cross-reactive MAbs, such as 4G2 and 6B6C-1 [53]. Thus, the EDII 101/106/107 region may be less likely to cooperate with the EDIII 329/331/389 region in inducing an antibody response when the immunogen been modified by formalin.

The formalin-inactivated, lactate dehydrogenase-elevating, virus-elicited antibodies differ from antibodies after natural infection. Formalin-inactivated influenza virus could not induce a T-cell response and was less protective in mice against homologous and heterologous influenza virus challenge as compared with γ-ray–inactivated virus [37,82]. However, another study indicated that the use of low formalin concentrations, short inactivation period, and high incubation temperature improved the immunogenicity of formalin-inactivated JEV vaccine and elicited high titers of neutralizing antibodies in mice [42]. Here, we showed that the binding activity of T16 MAb was reduced more by virus inactivation at 22°C than 4°C for the same treatment duration. Surprisingly, UV-inactivated Nakayama virus failed to be recognized by MHIAF and T16. Adjusting the condition for UV irradiation may maintain the antigenic structure of JEV. UV light inactivates virus by cross-linked viral nucleic acid and viral proteins. Cross-linked by oxidation between the amino acid residues may increase the susceptibility of protease cleavage [83,84], and degradation of aromatic side chain of amino acid and disulfide bond forming cysteine in protein has been indicated after UV treatment [85,86]. The loss of viral antigenicity was also observed in UV-inactivated virus including poliovirus (showing both antigenic and morphologic change), and influenza A virus (exhibiting low hemagglutination activity) [37,87]. Murray Valley encephalitis virus, belonging to JEV serocomplex, inactivated with UV showed lower immunogenicity compared to non-infectious VLP but the UV-induced antigenic change wasn’t described [88].

In conclusion, formalin and UV inactivation alter the antigenic structure of E protein in JEV and reduce the immunogenicity of associated vaccines. H_2_O_2_ inactivation seems to be a better alternative for JEV vaccine production. It maintained the antigenic structure of E protein, measured by a panel of MAbs. Further study should focus on identifying an optimal inactivation procedure and testing the immunogenicity of H_2_O_2_-inactivated JEV vaccine. Finally, to prevent unexpected modification of the various epitopes on the JEV vaccine during inactivation, a non-infectious JEV VLP or DNA vaccine should be developed. Formalin inactivation introduces an antigenic modification that affects the EDIII of JEV and thus distorts the profile of vaccine-induced neutralizing antibodies. Antigenic-stable inactivation methods are needed to develop better-inactivated JEV vaccines.

## Supporting Information

S1 Fig Monoclonal antibody (MAb) binding activity of formalin-inactivated virus (FIV) and untreated control virus (UCV) Japanese encephalitis viruses (JEVs). MAb binding activity of FIV- and UCV-SA14-14-2 (A), -T1P1 (B), and -YL2009-4 (C) viruses. Ag-ELISA of binding activities adjusted by antigen concentration according to OD_450_ of MHIAF, compared to day 0 (as 100%). Data are mean±SD of two duplicates, and the significant difference was indicated as an asterisk (*p*<0.05).
(PDF)

S2 Fig JEV wild-type (WT) and EDIII 329/331/389 virus-like particles (VLPs) expression plasmid-transformed COS-1 cells. COS-1 cells were transformed with 30μg plasmids and stained with Evans blue (panel a to c) and mouse anti-JEV HIAF (panel d to f) 24 hr later.
(PDF)

S1 Table Nucleotide sequences of primers used for plasmid-encoded–mutated JEV virus-like particles. (PDF)

S2 Table Epitope-specific antibody response in serum samples collected from FICV-immunized children. (PDF)
