# Supplementary figures and images for: Formalin Inactivation of Japanese Encephalitis Virus Vaccine Alters the Antigenicity and Immunogenicity of a Neutralization Epitope in Envelope Protein Domain III
Source: PLoS Negl Trop Dis. 2015 Oct 23;9(10):e0004167. doi: 10.1371/journal.pntd.0004167 (PMC4619746; doi:10.1371/journal.pntd.0004167)

A

SA14-14-2

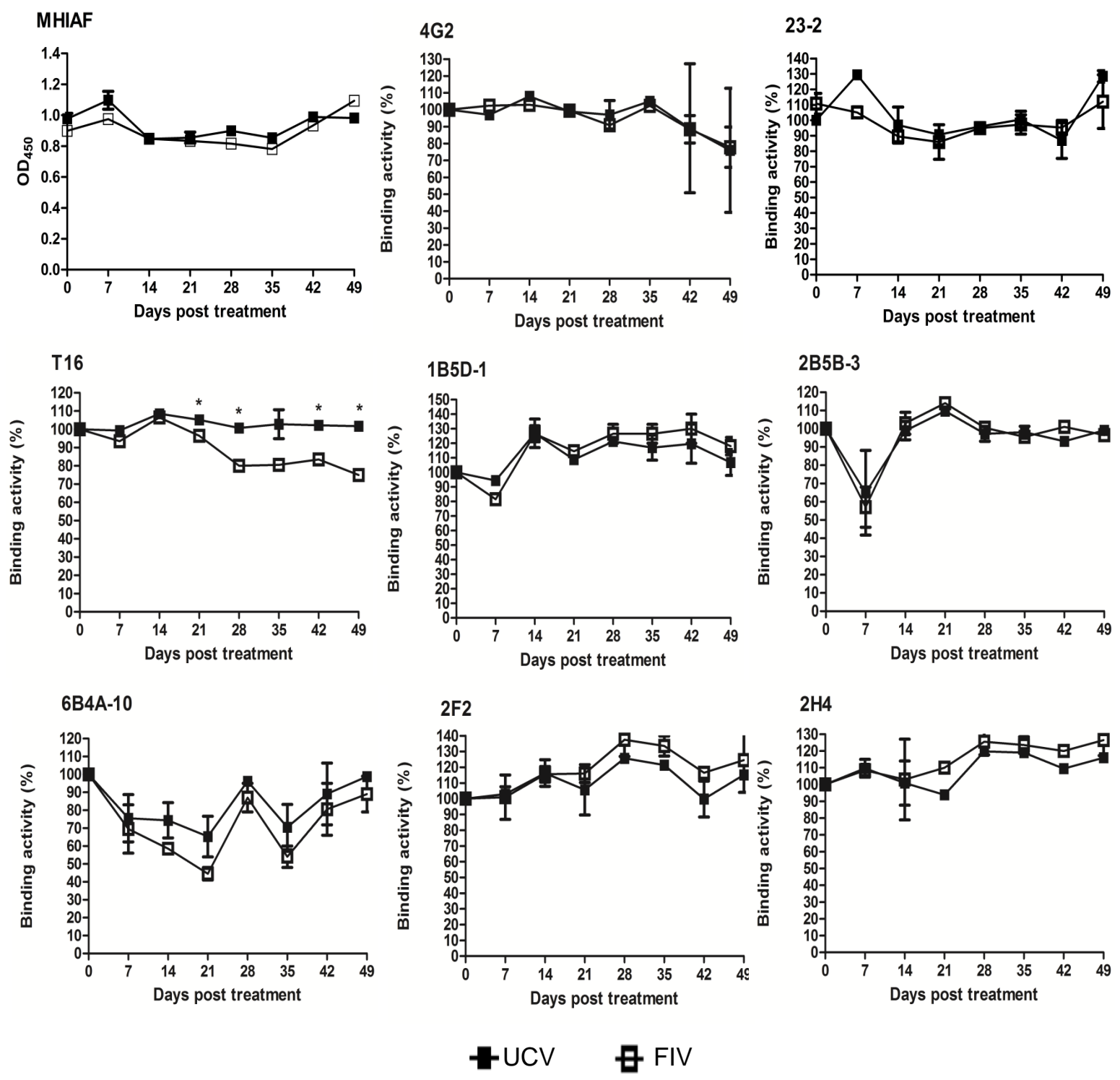

B

T1P1

MHAF

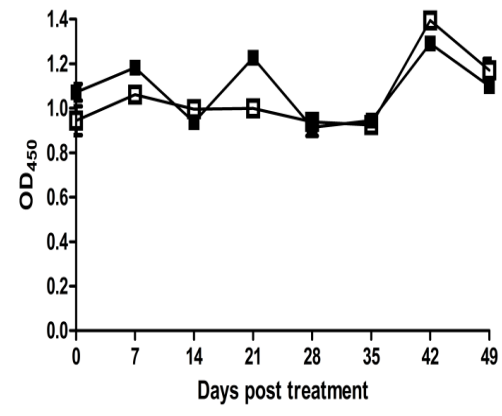

4G2

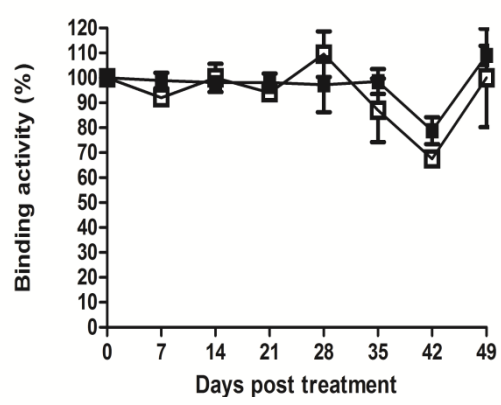

23-2

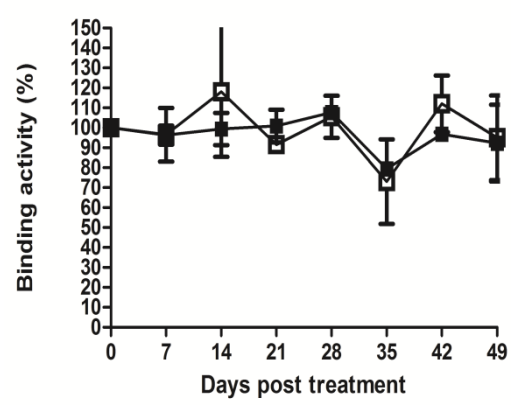

T16

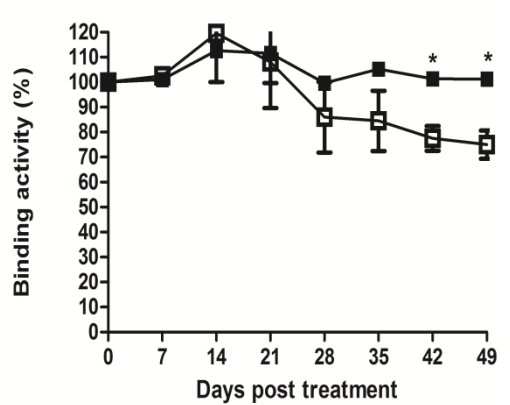

1B5D-1

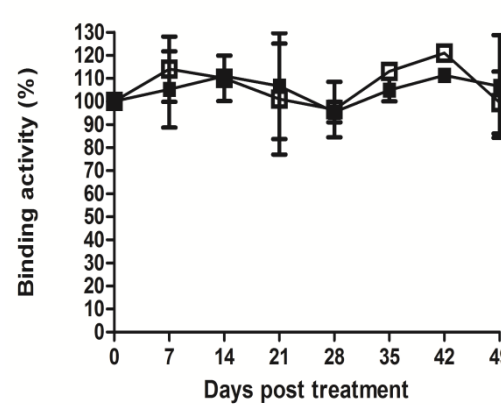

2B5B-3

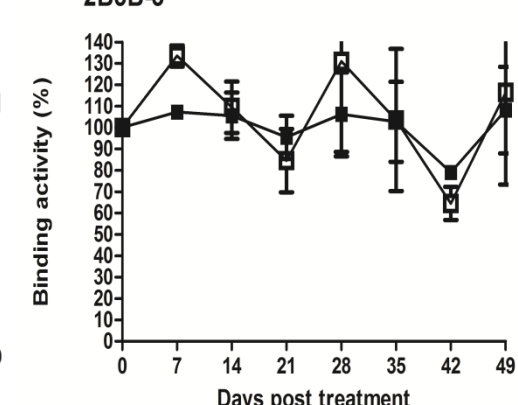

6B4A-10

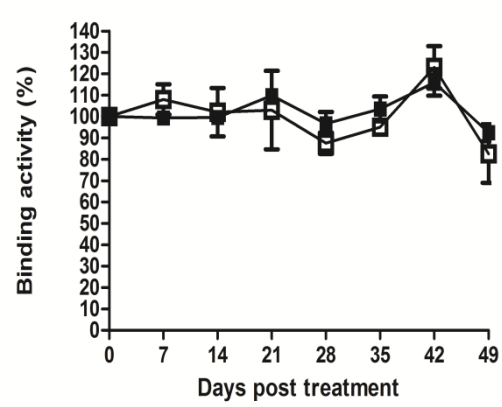

2F2

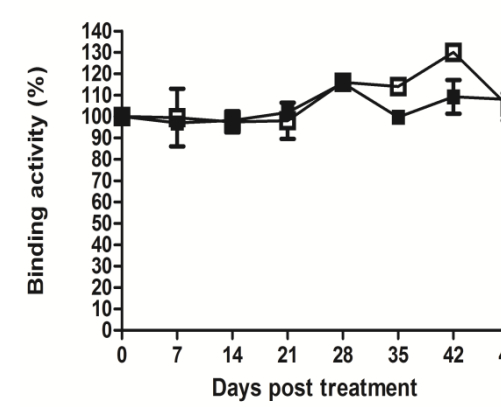

2H4

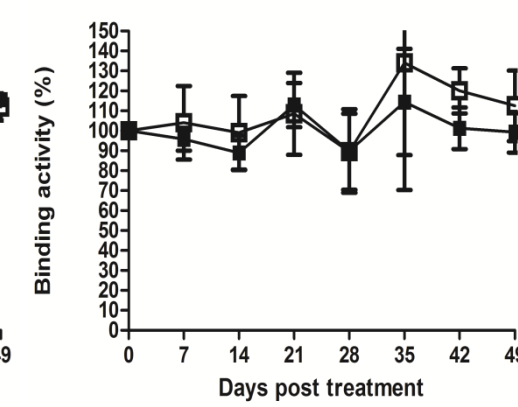

■ UCV    □ FIV

C

YL2009-4

MHIAF

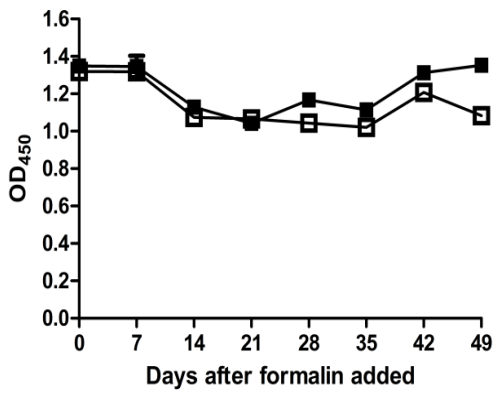

4G2

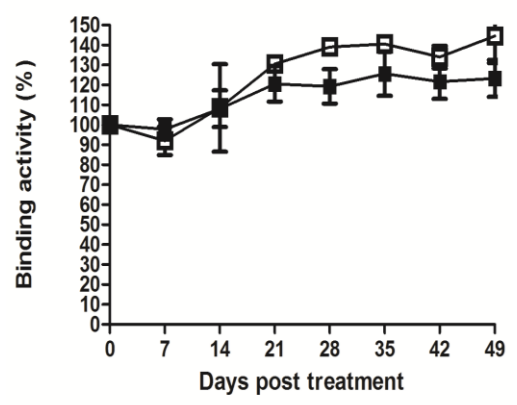

23-2

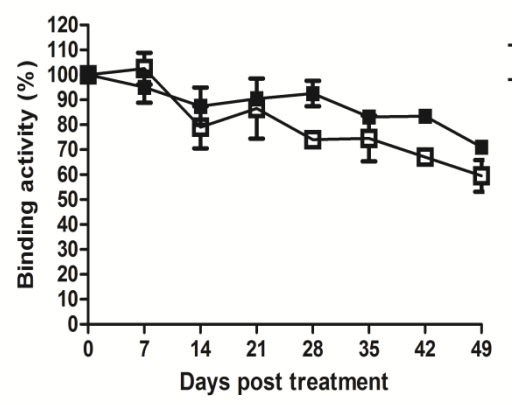

T16

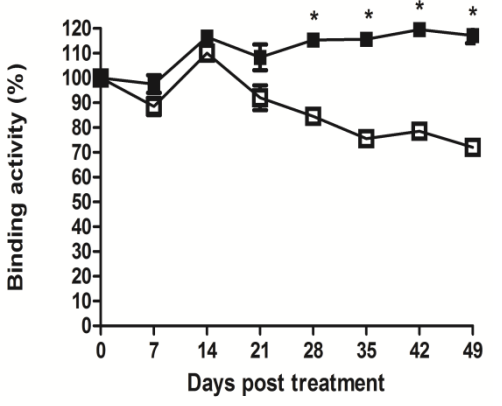

1B5D-1

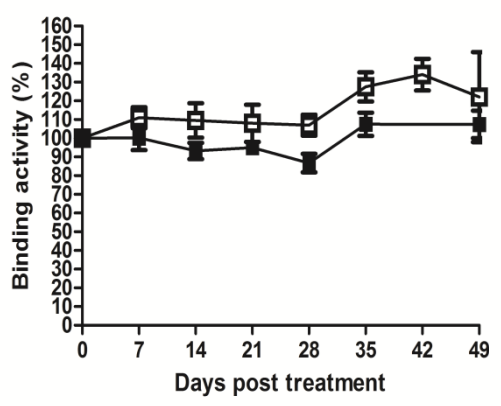

2B5B-3

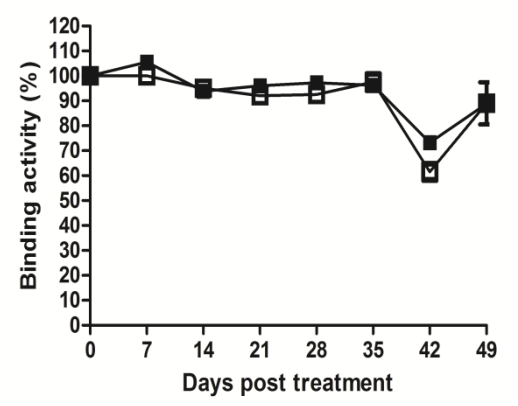

6B4A-10

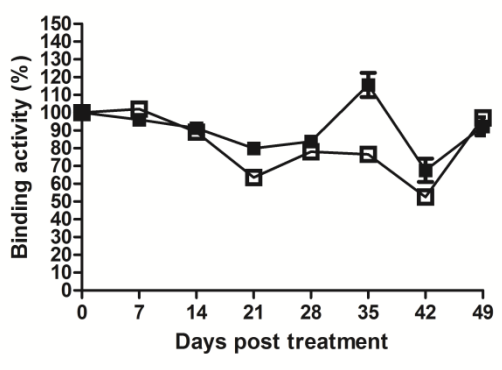

2F2

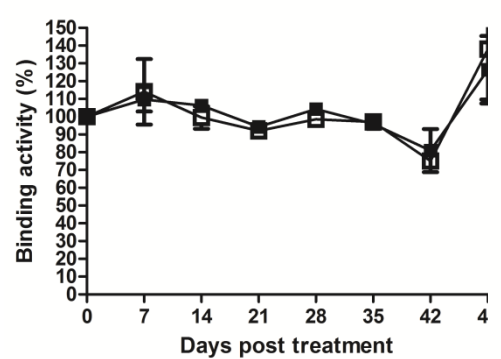

2H4

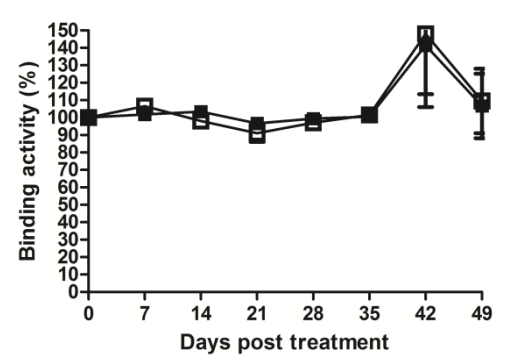

■ UCV    □ FIV

Supplement: S1 Fig — MAb binding activity of FIV- and UCV-SA14-14-2 (A), -T1P1 (B), and -YL2009-4 (C) viruses. Ag-ELISA of binding activities adjusted by antigen concentration according to OD450 of MHIAF, compared to day 0 (as 100%). Data are mean±SD of two duplicates, and the significant difference was indicated as an asterisk (p<0.05). (PDF) [file pntd.0004167.s001.pdf]

Evans blue

Mouse anti-JEV HIAF

(a)

(d)

Cell control

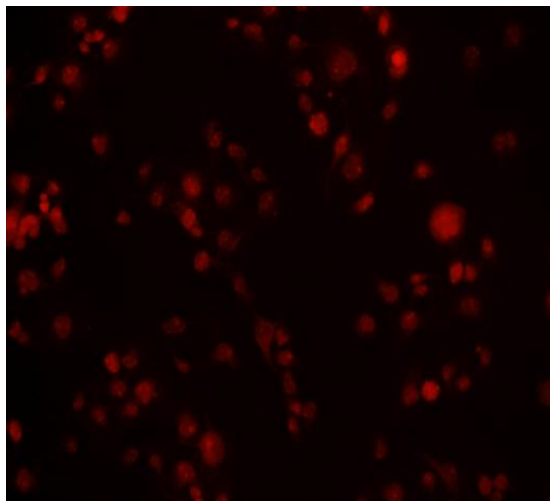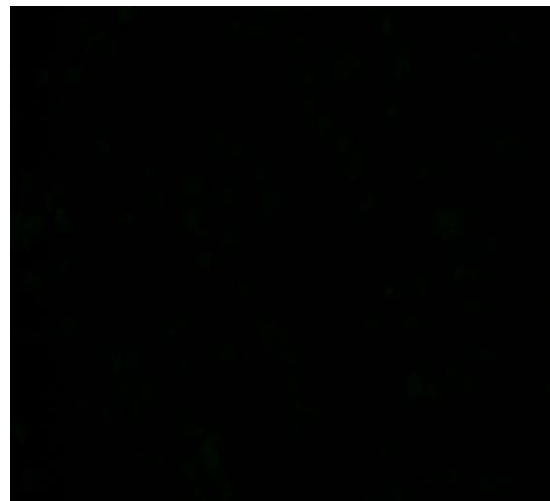

(b)

(e)

WT

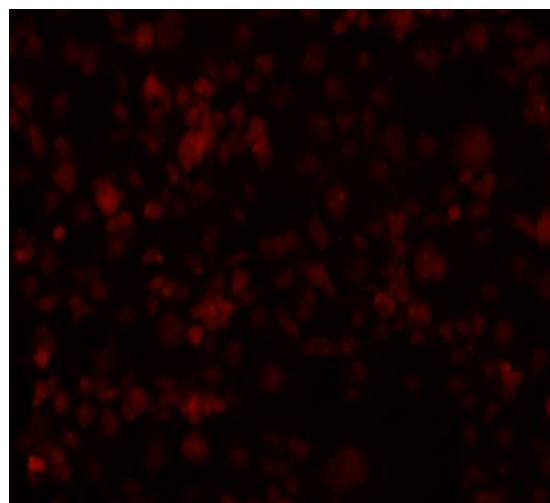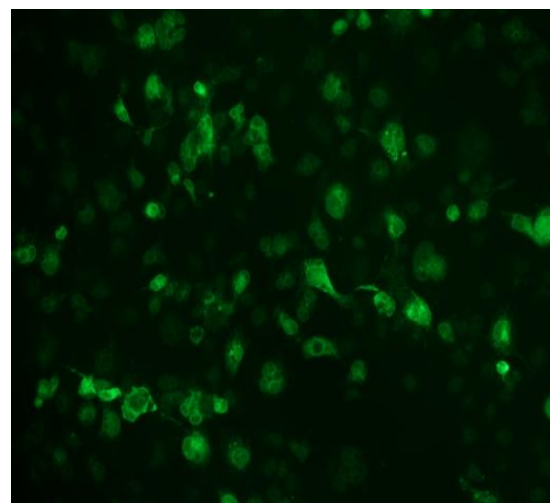

(c)

(f)

EDIII329/331/389

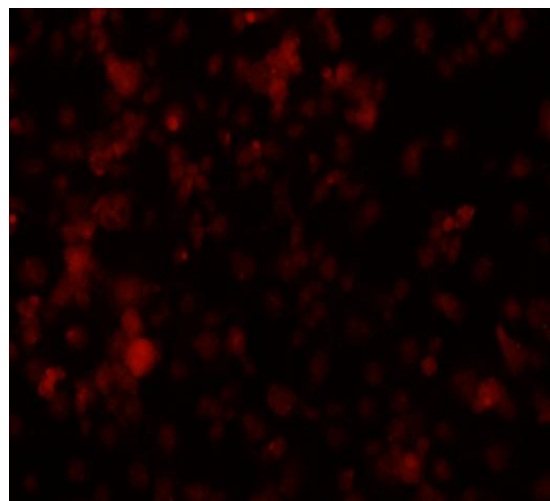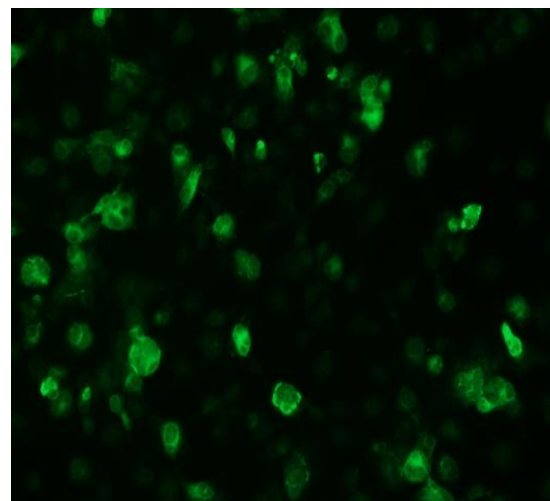

Supplement: S2 Fig — COS-1 cells were transformed with 30μg plasmids and stained with Evans blue (panel a to c) and mouse anti-JEV HIAF (panel d to f) 24 hr later. (PDF) [file pntd.0004167.s002.pdf]
