# Supplementary material for: Formalin Inactivation of Japanese Encephalitis Virus Vaccine Alters the Antigenicity and Immunogenicity of a Neutralization Epitope in Envelope Protein Domain III
Source: PLoS Negl Trop Dis. 2015 Oct 23;9(10):e0004167. doi: 10.1371/journal.pntd.0004167 (PMC4619746; doi:10.1371/journal.pntd.0004167)
Supplement: S1 Table — (PDF) [file pntd.0004167.s003.pdf]

S1 Table. Nucleotide sequences of primers used for plasmid-encoded–mutated JEV virus-like particles.

Primers	Length (nt)	Nucleotide sequences (5'-3')[a]	Nucleotide substitutions	Amino acid substitutions
W101G/G106K/L107D	45	CAAGCTTCCCTTCCCGAA**ATCTTT** ACATCCGTTGCC**TCC**CCCACG	TGG-GGA<br>GGA-AAA<br>CTT-GAT	Trp-Gly<br>Gly-Lys<br>Leu-Asp
E306G	38	TTTCGCGAACGAGAATTT**GCC**TGT ACACATGCCATAGG	GAA-GGC	Glu-Gly
S329A	36	GGGGCCATCACTCCC**GGC**GTAGG AGAGTTCAATGAC	TCT-GCC	Ser-Ala
S331K	36	TTTGCAGGGGCCATC**CTT**CCCAGA GTAGGAGAGTTC	AGT-AAG	Ser-Lys
D332R	36	TCGGAATTTTGCAGGGGCC**TCT**AC TCCCAGAGTAGG	GAT-AGA	Asp-Agr
D389G	36	GGTGGTTGATCTGCTT**TCC**TCCCC TTCCAACTACGA	GAC-GGA	Asp-Gly
S329A/S331K	36	TTTGCAGGGGCCATC**CTT**CCC**GGC** GTAGGAGAGTTC	TCT-GCC<br>AGT-AAG	Ser-Ala<br>Ser-Lys

[a] Mutated nucleotides are shown in boldface
