# Supplementary material for: Formalin Inactivation of Japanese Encephalitis Virus Vaccine Alters the Antigenicity and Immunogenicity of a Neutralization Epitope in Envelope Protein Domain III
Source: PLoS Negl Trop Dis. 2015 Oct 23;9(10):e0004167. doi: 10.1371/journal.pntd.0004167 (PMC4619746; doi:10.1371/journal.pntd.0004167)
Supplement: S2 Table — (PDF) [file pntd.0004167.s004.pdf]

S2 Table. Epitope-specific antibody response in serum samples collected from FICV-immunized children.

Source	no. of serum samples	GAC-ELISA		
		JEV antigen	Epitope-specific response (%)	
			Range	Mean
FICV-immunized children	12	WT	100	-
		EDII 101/106/107	36-88	61
		EDIII 329/331/389	10-46	23
